# Nanopore sequencing as a novel method of characterising anorexia nervosa risk loci

**DOI:** 10.1186/s12864-024-11172-7

**Published:** 2024-12-31

**Authors:** Natasha Berthold, Silvana Gaudieri, Sean Hood, Monika Tschochner, Allison L. Miller, Jennifer Jordan, Laura M. Thornton, Cynthia M. Bulik, Patrick Anthony Akkari, Martin A. Kennedy

**Affiliations:** 1 University of Western Australia, Crawley, WA Australia; 2 Perron Research Institute, Nedlands, WA Australia; 3 Pathology and Biomedical Science Department, University of Otago Christchurch, Christchurch, New Zealand; 4 Murdoch University, Murdoch, WA Australia; 5 University of Notre Dame Australia, Fremantle, WA Australia; 6 Vanderbilt University Medical Centre, Nashville, TN USA; 7 Department of Psychological Medicine, University of Otago Christchurch, Christchurch, New Zealand; 8 Department of Psychiatry, University of North Carolina at Chapel Hill, Chapel Hill, USA; 9 Department of Medical Epidemiology and Biostatistics, Karolinska Institutet, Stockholm, Sweden; 10 Department of Nutrition, University of North Carolina at Chapel Hill, Chapel Hill, USA; 11 Duke University, Durham, NC USA

**Keywords:** Eating disorders, Psychiatric genetics, Anorexia nervosa risk loci, Structural variants, Transposable elements, Nanopore sequencing, Long-read sequencing, Adaptive sampling, Targeted sequencing

## Abstract

**Background:**

Anorexia nervosa (AN) is a polygenic, severe metabopsychiatric disorder with poorly understood aetiology. Eight significant loci have been identified by genome-wide association studies (GWAS) and single nucleotide polymorphism (SNP)-based heritability was estimated to be ~ 11–17, yet causal variants remain elusive. It is therefore important to define the full spectrum of genetic variants in the wider regions surrounding these significantly associated loci. The hypothesis we evaluate here is that unrecognised or relatively unexplored variants in these regions exist and are promising targets for future functional analyses. To test this hypothesis, we implemented a novel approach with targeted nanopore sequencing (Oxford Nanopore Technologies) for 200 kb regions centred on each of the eight AN-associated loci in 10 AN case samples. Our bioinformatics pipeline entailed base-calling and alignment with Dorado and minimap2 software, followed by variant calling with four separate tools, Sniffles2, Clair3, Straglr, and NanoVar. We then leveraged publicly available databases to characterise these loci in putative functional context and prioritise a subset of potentially relevant variants.

**Results:**

Targeted nanopore sequencing effectively enriched the target regions (average coverage 14.64x). To test our hypothesis, we curated a list of 20 prioritised variants in non-coding regions, poorly represented in the current human reference genome but that may have functional consequences in AN pathology. Notably, we identified a polymorphic SINE-VNTR-Alu like sub-family D element (SVA-D), intergenic with *IP6K2* and *PRKAR2A*, and a poly-T short tandem repeat (STR) in the 3ʹUTR of *FOXP1*.

**Conclusions:**

Our results highlight the potential of targeted nanopore sequencing for characterising poorly resolved or complex variation, which may be initially obscured in risk-associated regions detected by GWAS. Some of the variants identified in this way, such as the polymorphic SVA-D and poly-T STR, could contribute to mechanisms of phenotypic risk, through regulation of several neighbouring genes implicated in AN biology, and affect post-transcriptional processing of *FOXP1*, respectively. This exploratory investigation was not powered to detect functional effects, however, the variants we observed using this method are poorly represented in the current human reference genome and accompanying databases, and further examination of these may provide new opportunities for improved understanding of genetic risk mechanisms of AN.

**Supplementary Information:**

The online version contains supplementary material available at 10.1186/s12864-024-11172-7.

## Introduction

Anorexia nervosa (AN) is a heritable, potentially lethal psychiatric disorder, lacking effective pharmacotherapeutic treatment. Twin studies estimate genetic variation accounts for ~ 48–74% of phenotypic variability [1] and a landmark genome-wide association study (GWAS) identified eight genetic loci significantly associated with AN [2]. However, underlying mechanisms of AN genetic risk remain elusive.

In addition to single nucleotide variants, structural genetic variants (SVs) are important contributors to polygenic risk. SVs are broadly classed by length, composition, and genomic position. SVs have been associated with pathogenic and biological mechanisms, including accessibility of genetic regulatory regions, expression efficiency, chromatin formation, and three-dimensional structure interactions, such as topological associating domains [3–6].

To date, many types of SVs have been difficult to capture by PCR-based and short-read sequencing techniques [4, 7–9], often resulting in exclusion from or poor characterisation in datasets and linear human reference genomes [10]. Recent application of long-read sequencing approaches, as well as identification and characterization of many SVs, has resulted in landmark global human genome efforts such as the Telomere-to-Telomere (T2T) project, and the Human Pangenome Reference Consortium (HPRC) [11, 12]. Here, we focus on prioritising characterisation of short structural variants, namely insertion/deletion (indel) elements, tandem repeat (TR) elements, as well as transposable elements, which can vary in size up to thousands of base pairs in length. High mutability and variety of putative biological impacts make these interesting candidates in contributing to AN heritability.

Nanopore sequencing (Oxford Nanopore Technologies; ONT) is a well-established long-read sequencing technique. It specifically has several attributes that make it useful for interrogating SVs and it is increasingly being used for this purpose [9, 13]. The high accuracy and long reads allow SVs many kilobases (kb) in length to be captured and characterized with single molecule resolution of haplotypes. Moreover, ONT devices allow enrichment of specific genomic regions by targeted sequencing, also known as adaptive sampling, where molecules that do not match a predetermined list of target regions are ejected from pores by current reversal [14]. This decreases sequencing time, reagent costs, and increases the read efficacy. Finally, targeted nanopore sequencing using this method can also capture native methylation data for the sequenced regions [15].

We hypothesise that part of AN heritability will be attributable to SVs in risk loci identified by GWAS [2], driving or contributing to the signal of strongly associated SNPs via linkage disequilibrium (LD) [7]. Consequently, detailed genomic characterization of the regions surrounding the eight AN GWAS-associated loci may reveal such variants and open new avenues to understanding the biological underpinnings of AN. We have previously proposed that SVs, specifically short tandem repeats (STRs), contribute to AN heritability [16]. Here, to explore this idea further, we use long-read nanopore sequencing technology to characterize the genetic architecture in eight AN risk regions [2] and apply a bioinformatics pipeline to prioritise SVs of interest for further investigations.

## Methods

**Fig. 1:**
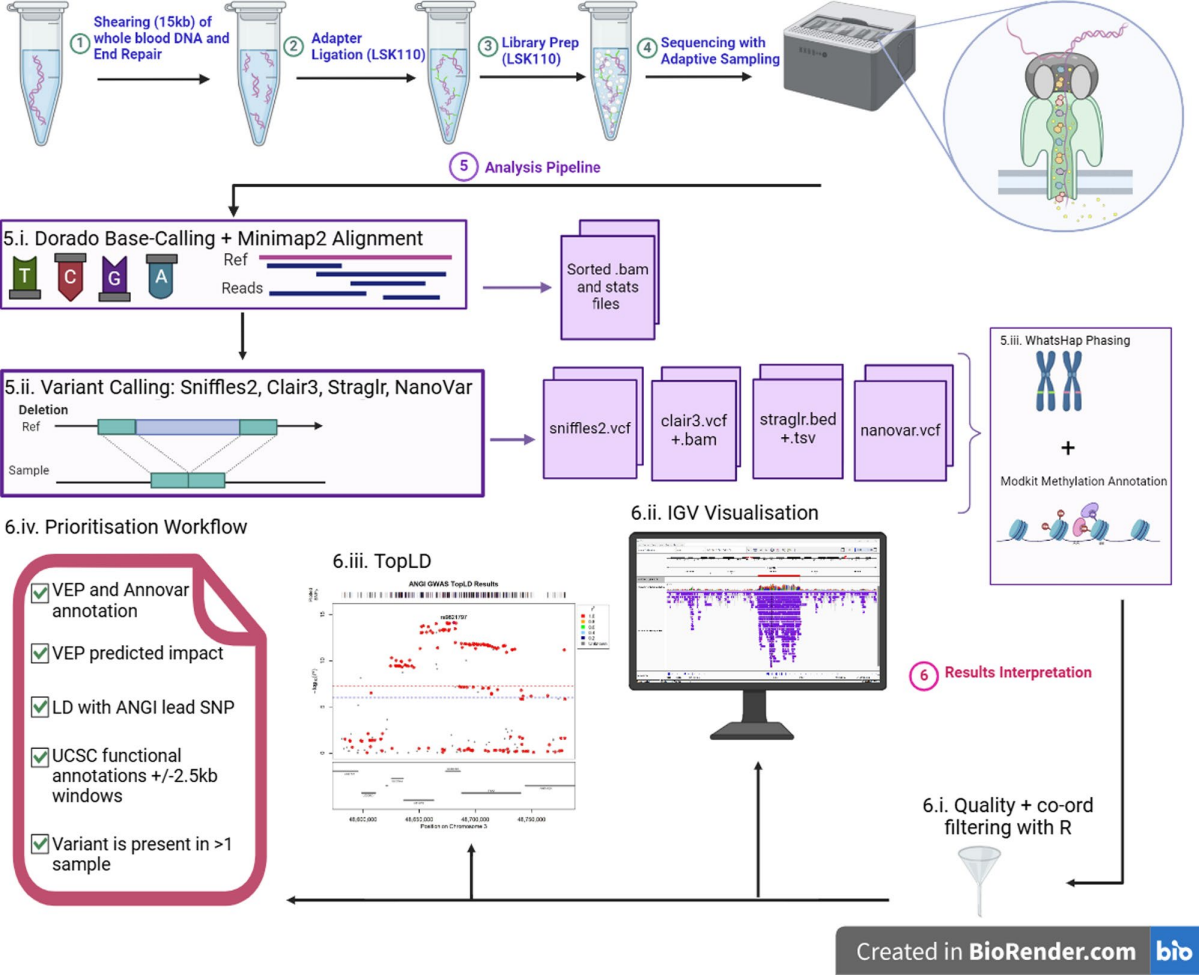
Workflow used to fine-map genomic regions surrounding the eight SNPs associated with anorexia nervosa in the ANGI GWAS [2]. Steps 1–4 represent steps of library preparation and sequencing with ONT’s ligation sequencing kit 110 (LSK110) protocol. The circular panel in step 4 is a cartoon of a DNA strand being ratcheted through a single nanopore. Step 5 shows the primary data analysis steps, including base-calling and alignment of the raw sequencing data, outputting sorted BAM and statistics files, followed by variant calling with four different callers- Sniffles2, Clair3, Straglr, and NanoVar, outputting their respective file types, VCF, VCF and BAM, BED and TSV, and VCF, respectively. Step 6 describes results interpretation, including filtering with R, IGV visualization of data, calculation of LD with TopLD, and prioritization workflow. Abbreviations: Kb - kilobases, Ref - reference, stats - statistics, co-ord - coordinates, IGV - Integrative Genomics Viewer, VEP - variant effect predictor, LD - linkage disequilibrium, UCSC - University of California Santa Cruz, SNP - single nucleotide polymorphism. Created with BioRender.com

We applied targeted nanopore sequencing to characterize the genetic architecture at base pair resolution of the eight AN risk regions (Table 1) in an exploratory case cohort (*n* = 10) drawn from Anorexia Nervosa Genetics Initiative (ANGI) [2]. The complete workflow is depicted in Fig. 1.

## Sample processing

### Samples and library preparation

The pilot cohort (*n* = 10) was a subsample of previously de-identified samples from the New Zealand participants in the ANGI who participated with informed consent, under approval from the Southern Health and Disability Ethics Committee (New Zealand) reference 14/STH/115. Original data collection and processing is described elsewhere [17]. Selected samples were all female participants with European ancestry.

Library preparation and sequencing were conducted as described in the manufacturer’s protocol and are depicted in Fig. 1. Briefly, libraries were prepared using Ligation Sequencing Kit (SQK-LSK110; ONT, UK), and DNA end repair and adapter binding preparation and adapter ligation were performed with NEBNext^®^ Companion Module for Oxford Nanopore Technologies^®^ Ligation Sequencing (E7180S; New England BioLabs, USA). Following DNA end repair and preparation, samples were subjected to a clean-up with MAGBIO beads (MAGBIO Genomics Inc, USA), prior to adapter ligation, as per ONT recommendations. Throughout the subsequent library preparation, DNA sample quality was assessed with a Qubit^®^ 2.0 Fluorometer (Thermofisher Scientific, USA).

### Programable target selection

Targeted sequencing was applied to the regions surrounding the eight genetic loci identified in the parent ANGI study [2] (Table 1). This was performed using the Read Until interface imbedded in the GridION software. Of note, the ANGI GWAS was based upon GRCh37, while our analyses were based upon GRCh38. Where conversion was required, we used the University of California Santa Cruz (UCSC) web browser tool LiftOver [18]. The target regions were 200 kb in length, centred on each lead ANGI GWAS SNP and named by region (see Table 1). Region size was selected as a compromise between detecting SVs of interest and computational burden of variant prioritisation without statistical power. A reference index specific to the target regions using the start and end co-ordinates was created with the Bedtools command line tool (v2.30.0) (Table 1) [19] with Ensembl’s GRCh38 reference genome and a BED file containing the respective target’s GRCh38 co-ordinates. This generated a reference FASTA file containing the sequences of all 200 kb target regions to be supplied to the GridION graphic user interface at sequencing. We performed a sequence BLAST on the target FASTA via the NCBI BLAST + command line tool (v2.13.0) [20] as a quality control check, to ensure that the target FASTA mapped to the correct target regions.

**Table 1:** Target regions for targeted nanopore sequencing

ANGI Lead SNP^1^	Chrm	Lead SNP Location	Gene(s)^2^	Target Region Name	Target Region Coordinates^3^
rs9821797	3	48,680,820	*NCKIPSD*	Target Region 1	48,580,820 − 48,780,820
rs6589488	11	115,226,236	*CADM1*	Target Region 2	115,126,236 − 115,326,236
rs2287348	2	53,812,676	*ASB3*, *ERLEC1*	Target Region 3	53,712,676 − 53,912,676
rs2008387	10	129,650,500	*MGMT*	Target Region 4	129,55,0500 − 129,750,500
rs9874207	3	70,970,599	*FOXP1*	Target Region 5	70,870,599 − 71,070,599
rs10747478	1	96,435,899	*PTBP2*	Target Region 6	96,335,899 − 96,535,899
rs370838138	5	25,081,736	*CDH10*	Target Region 7	24,981,736 − 25,181,736
rs13100344	3	94,886,263	*NSUN3*	Target Region 8	94,786,263 − 94,986,263

^1^ rsID of the most significant SNP from Watson et al. (2019)

^2^ Nearest coding gene to the lead SNP

^3^ GRCh38 coordinates; each selected region includes 100 kb either side of the lead SNP, resulting in 200 kb target region

## Sequencing

Targeted sequencing was conducted with MinION flow cells R9.4.1 (ONT) on the GridION platform (ONT). Flow cells were primed with Flow Cell Priming Kit (LSK110; ONT). Samples were sequenced for a total duration of ~ 72 h, or until flow cell exhaustion. During sequencing, reads < Q9 and < 500 bp were automatically removed. To maximise sequencing efficacy, nuclease flushes and sample reloading were performed at ~ 24 and ~ 48 h. Raw data was output in POD5 format.

## Bioinformatics analysis

A bioinformatics pipeline (Fig. 1) was implemented for data analysis. Briefly, post-sequencing base-calling of raw data was performed with Dorado (v0.5.0), using model dna_r9.4.1_e8_sup@v3.3_5mCG_5hmCG@v0, modified bases selected, and Minimap2 alignment to GRCh38. Data was generated as BAM files, then indexed with Samtools for variant calling.

To better capture the spectrum of genetic variation, variant calling was performed with four different callers, Sniffles2 (v2.2) [21], Straglr (v1.4.1) [22], Clair3 (v1.0.4) [23], and NanoVar (v1.5.0) [24]. This was followed by allele phasing and haplotagging with WhatsHap (v1.7); [25]. To eliminate potential errors within the pipeline, we performed a benchmark with raw sequencing data and benchmark variant call sets from the publicly available and highly curated Genome in a Bottle sample HG002 [26].

Variant call and alignment outputs were visualised with Integrative Genomics Viewer (IGV) desktop application (v2.1.6.0) [27] against IGV annotation tracks. Variants were filtered to contain only variants in the target regions (Table 1) and variant quality score threshold of >/=9, to remove spurious calls. Following this filtering, functional annotation tools ANNOVAR [28] and Ensembl Variant Effect Predictor (VEP) [29] were used to obtain basic annotation information for all variants and aid prioritisation of a subset potentially relevant to AN pathobiology. VEP was used to annotate the impact of variant alleles input from the three variant calling algorithms that output VCFs on transcripts from Ensembl/GENCODE and RefSeq databases.

We developed a prioritisation pipeline for identifying structural variants of putative interest (Fig. 1, step 6.iv) from all variants output by the calling algorithms. First, we removed all SNVs and focused on short structural variants, such as indels > 10 bp in length, tandem repeats, and transposable elements likely to have poorly resolved genotypes in reference data bases. Consequently, we did not specifically look for variants based on rarity. Second, we used the VEP functional annotations to filter for variants with “High” impact, meaning non-reference alleles had predicted functional consequences on the respective transcript, and no existing reported variant rsID to improve likelihood of capturing under characterised variants. While the study was underpowered to calculate LD between the SVs genotyped and ANGI SNPs, we interrogated LD from the ANGI GWAS summary statistics within our regions with TopLD [30]. This produced a graph depicting the LD between SNVs in the region and the lead ANGI hit, as well as significance values for those SNVs from the GWAS. Variants overlapping blocks of high LD with the significant ANGI GWAS SNPs were retained as most likely to be contributing to the GWAS signal; given the weaker GWAS signal, a more lenient significance threshold for regions of LD in non-coding target regions 6–8 was chosen and trends towards significance were considered (Supplementary Table 1; Supplementary Fig. 3 materials). We manually inspected variants, comparing across callers, BAM files, and samples to determine allele diversity, concordance, and identify how well the different callers were able to resolve variants. Due to the exploratory nature of our study, we considered variant presence or absence, rather than consensus genotype. A variant was present for a sample if called by at least one of the four variant callers, or the BAM files clearly differed from the reference genome on visual examination. Variation was defined as a difference in length or structure, to better capture a spectrum of variation. Additionally, we used UCSC Genome Browser tracks for GRCh38 to identify potentially functional annotations not captured in the earlier VEP and ANNOVAR analyses, within +/-2.5 kb of the variant, a plausible effect window for short structural variants, tandem repeats, and transposable elements. Finally, we compared prioritised variants to the 90 HPRC assembly tracks available on the UCSC Genome browser allele frequencies [11, 31, 32].

All command line tools were implemented in batch script on the University of Western Australia’s Centos8 Kaya high performance computer cluster. We performed all file manipulation in R (v4.3.3).

## Results

We performed a targeted nanopore sequencing proof-of-principle study in a small cohort of AN cases (*n* = 10) and successfully characterised the regions surrounding the eight AN risk loci, at nucleotide resolution. Target regions were clearly enriched in the sequence data obtained, relative to off-target regions, showing higher average read lengths and coverages (Table 2; Supplementary Fig. 1).

**Table 2:** Targeted nanopore sequencing base-calling and alignment statistics

		On Target Reads			Off Target Reads		
	Sample	Avg Length^1^	Avg Qual^2^	Avg Cov^3^	Avg Length^1^	Avg Qual^2^	Avg Cov^3^
	S1	8216	22.5	10.4	3091	21	2.8
	S2	8470	22.6	13.4	3195	22.2	2.9
	S3	9538	21.3	16.4	3192	22.6	3.4
	S4	12,148	22.6	17.7	2745	22.5	3.6
	S5	12,379	22.4	10.3	2678	22.6	2.2
	S6	14,554	22.2	18.6	1968	22.5	3.7
	S7	14,763	22.2	13.5	2407	22.5	4.0
	S8	14,774	22	18.4	4021	23	4.6
	S9	15,169	22.5	15.8	3625	22.2	4.7
	S10	15,400	22.9	11.9	3609	22.1	3.5
SE^4^		903.90	0.14	1.00	195.63	0.17	0.25

^1^Average length of the read in base pairs

^2^Average quality of reads calculated based on error probability. The threshold score is 9

^3^Average coverage is the average number of reads that map over a base pair

^4^Standard error calculations

A total of 22,380 variants were detected from the combined VCF outputs of Sniffles2, Clair3, NanoVar, and Straglr, for all eight target regions and 10 samples. This included a total of 17,328 SNVs and 5052 non-SNV SVs (Supplementary Fig. 2a-i; Supplementary Tables 2 and 3). The average frequency of non-SNVs per sample was 0.226 (S.E. 0.004). The VEP basic annotation report annotated both SNVs and non-SNVs captured by Clair3, Sniffles2, and NanoVar (*n* = 18,980). The report indicated that as expected most (52%) variants were intronic, followed by non-coding transcript variants (24%), and the remaining 24% comprised downstream, upstream, intergenic, nonsense mediated decay transcripts, regulatory region, noncoding transcript exon, transcription factor binding site (TFBS) and other (Supplementary Fig. 2a). VEP was unable to associate any of the non-SNVs with existing variant rsID s, and therefore annotated these as “novel”. The significance of regions of LD in the TopLD graphs decreased as the significance of the ANGI GWAS SNP decreased (Supplementary Fig. 3).

**Fig. 2:**
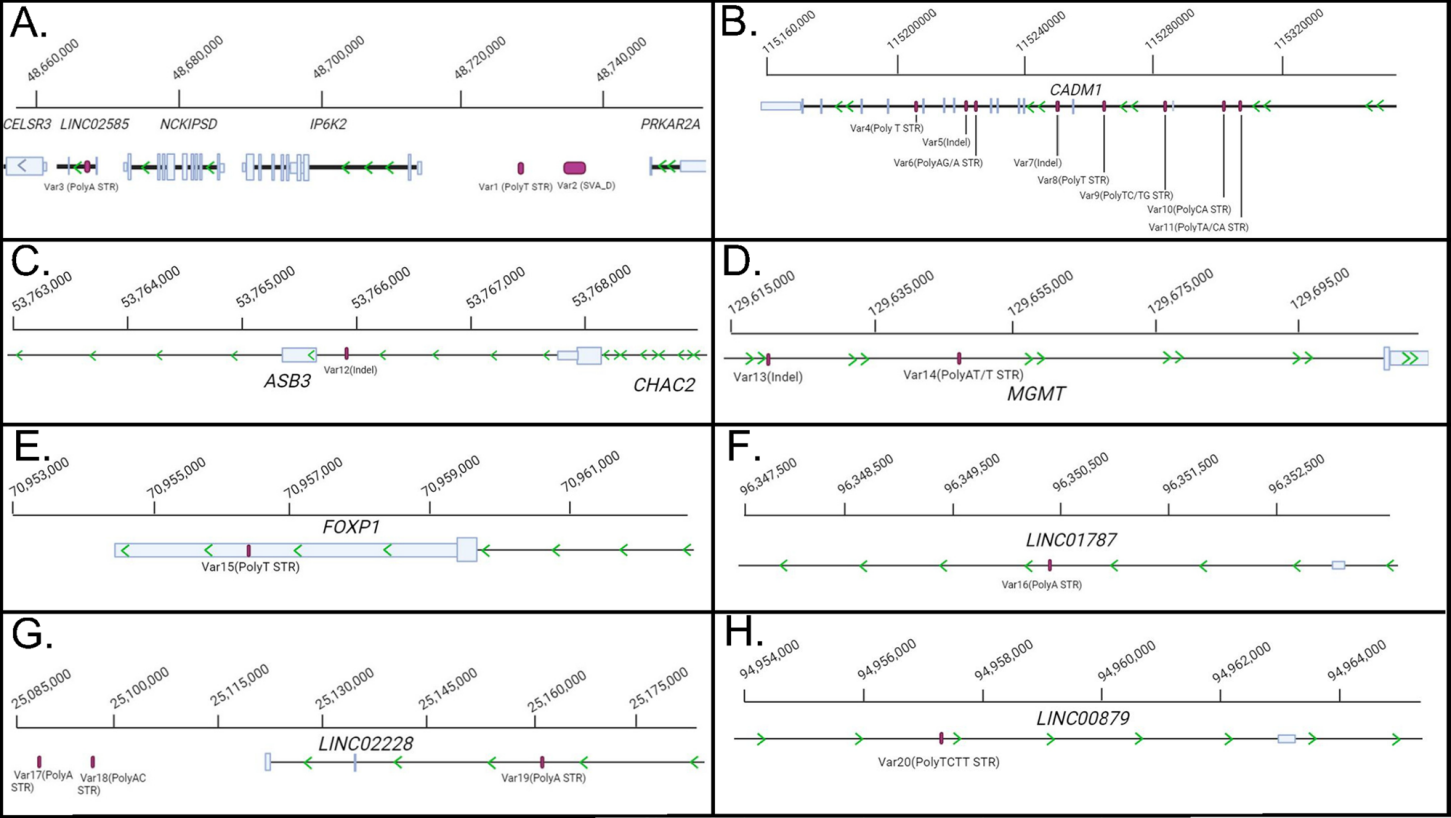
Partial gene maps of the eight target regions, annotated with the 20 prioritised variants. The panels are zoomed in from the 200 kb regions to focus on the prioritised SVs. Green arrows show transcriptional orientation. (**A**) Target region 1 showing Var1 and Var2 intergenic to genes *IP6K2* and *PRKAR2A*, and Var3, intronic to non-coding element *LINC02585*. (**B**) Target region 2, with variants Var4-Var11 overlapping gene *CADM1*. (**C**) Target region 3, showing Var12 intronic to gene *ASB3* and upstream of gene *CHAC2*. *ASB3* and *CHAC2* are on complimentary DNA strands. (**D**) Target region 4 showing gene *MGMT* and the intronic variants Var13 and Var14. (**E**) Target region 5, showing Var15 overlapping the 3ʹUTR of gene *FOXP1*. (**F**) Target region 6, showing Var16 intronic to the non-coding element *LINC01787*. (**G**) Target region 7, showing variants Var17-Var19 proximal and intronic to non-coding element *LINC02228*. (**H**) Target region 8, showing Var20 intronic to non-coding element *LINC00879*

We focused on the 5052 non-SNV variants to prioritise those that were poorly characterised in large databases and therefore more likely to be initially cryptic to GWAS. We used the prioritisation criteria outlined in Methods and selected a subsample of 20 variants (named Var1-20) for further investigation (Table 3; Fig. 2). All samples showed non-reference alleles for variants Var1-4, 6, 8–10, 12–20. A total of three, six, and seven samples showed non-reference alleles for Var5, Var7, and Var11, respectively. The three indel variants (Var5, 7, 12 and 13) had the most concordant allele genotypes across the four different calling algorithms and BAM files, while the 15 STRs and the *SINE-VNTR-Alu* subfamily D (SVA-D) element all showed greater discordance (Table 3; Supplementary Tables 5 and 6). Most variants occurred in non-coding regions, particularly intronic regions, with one (Var20) annotated as non-coding exon (Fig. 2).

**Table 3:** Characteristics of variants prioritized by bioinformatic pipeline

ID	Chr	Start(Ref)	End(Ref)	SV Type	Ref Sequence	Sniffles2	NanoVar	Clair3	Straglr	BAM
Var1	3	48,729,044	48,729,084	Poly-T	(T)_40_	4(4)				All
Var2	3	48,733,473	48,735,453	SVA-D	(CCCTCT)_n_(Alu-like)(VNTR)(SINE-R)(A)_n_	7(3)	4(3)	5(3)	10(48)^1^	All
Var3	3	48,668,100	48,668,118	Poly-A	(A)_19_	1(1)				All
Var4	11	115,206,759	115,206,789	Poly-T	(T)_31_	1(1)				All
Var5	11	115,222,603	115,222,618	Indel (Deletion)	ATAGTACTGAAACTGG	3(1)		3(1)		3
Var6	11	115,223,959	115,223,996	Poly-A/AG	(A)_16_(AG)_11_	1(1)				All
Var7	11	115,251,423	115,251,457	Indel (Deletion)	ACTGACATTACTACAGAAACAGAAAATTGGGAAG	6(2)	2(1)	5(1)		6
Var8	11	115,267,675	115,267,698	Poly-T	(T)_3_C(T)_21_	1(1)				All
Var9	11	115,284,088	115,284,155	Poly-CT/GT	(CT)_14_/(GT)_14_	7(10)		1(1)	10(20)	All
Var10	11	115,306,071	115,306,105	Poly-AC	(AC)_17_	8(7)		5(2)	10(19)	All
Var11	11	115,308,195	115,308,216	Poly-TA/CA	(TA)_9_(CA)_2_	1(1)		6(4)		7
Var12	2	53,765,899	53,765,957	Indel (Deletion)	(TCTTTGGTGCTCCATTGATAAGAGCACC)_2_	5(4)	3(2)	3(1)	8(4)	All
Var13	10	129,618,768	129,618,773	Indel-(T)(+ 3 bp)(T)(T < A)(T)(+ 48–50 bp)	TTTTA	9(3)	6(3)	4(2)		All
Var14	10	129,646,720	129,646,755	Poly-AT/T	(AT)_18_(T)_3_	5(7)	1(1)	4(6)	10(9) (Poly AT only)	All
Var15	3	70,956,733	70,956,773	Poly-T	(T)_41_	2(2)				All
Var16	1	96,350,472	96,350,492	Poly-A	(A)_21_	2(2)				All
Var17	5	25,088,850	25,088,920	Poly-AAAAT	(AAAAT)_14_	8(4)	3(1)	8(4)	10(9)	All
Var18	5	25,097,623	25,097,671	Poly-AC	(AC)_22_ (imperfect)	3(1)		4(4)	10(10)	All
Var19	5	25,160,644	25,160,688	Poly-AC	(AC)_22_	3(4)			10(10)	All
Var20	3	94,957,289	94,957,431	Poly-TCTT	(TCTT)_21_	5(5)		2(2)	10(31)	All

Twenty variants were prioritized from amalgamated variant calling and annotation software, across all 10 samples. Columns 1–6 list study specific identifier, chromosome number, GRCh38 start position, GRCh38 end position, structural variant type, and reference sequence, respectively. For SV type: ‘Indel’ refers to insertion/deletion; Short tandem repeat variants are denoted by Poly-motif, with motif units comprised of thymine (T), adenosine (A), guanine (G), or cytosine (C); SVA-D is a SINE-VNTR-Alu retrotransposon of subfamily D, where SINE is short interspersed nuclear element, and VNTR is variable number tandem repeat. Columns 7–11 list the number of samples that the variant occurred in, followed by the number of alternative alleles in brackets that were observed, in each of the variant callers (Sniffles2, NanoVar, Clair3, Straglr) and BAM files

^1^Straglr did not identify the SVA element as an entire unit, so each tandem repeat identified across the SVA region was counted individually

### Prioritised SV structural characteristics

Discerning consensus genotypes for the prioritised SVs was challenging as both call rate and genotyping differed between variant calling algorithms. Three variants occurred in target region 1, a poly-T STR (Var1), an SVA-D (Var2) retrotransposon, and a poly-A STR (Var3; Fig. 2a). Both Var1 and Var2 were intergenic between *PRKAR2A* and *IP6K2*. Non-reference alleles of Var1 were all contractions. Var2 had a more complex repeat structure, with a (CCCTCT)n STR head, followed by an Alu-like element, and VNTR, a SINE-R element, and finally a (A)n STR tail. Straglr identified three different subunits in the (CCCTCT)n STR head and did not provide a clear consensus genotype for the region in any sample (Supplementary Table 6). Similarly, Straglr also identified seven different subunits within the VNTR (Supplementary Table 6). Conversely, Sniffles2, NanoVar, and Clair3 reported insertions in the VNTR region only (Supplementary Table 5). NanoVar also reported a 47 bp insertion (*n* = 3) and a 46 bp insertion (*n* = 1). Sniffles reported three different insertions, 38 bp (*n* = 1), 46 bp (*n* = 1), and 47 bp (*n* = 5). Finally, Clair3 reported only a 47 bp insertion (*n* = 5). None of the four variant callers recognised it as an entire element. Interestingly, another SVA-D element occurred just proximal to target region 1, *SHISA5* and showed sufficient coverage to indicate similar variation to Var2. Var3 was intronic to *LINC02585* and overlapped the poly-A tail of a transposable AluY element. It was only detected by Sniffles in Sample 7, but the algorithm was unable to resolve a genotype and therefore classed it as homozygous reference. Inspection of individual reads for Sample 7 showed 13 total reads, with five different insertions (1 bp, 2 bp, 3 bp, 15 bp, 16 bp), five deletions (2 × 1 bp, 2 bp, 4 bp, 8 bp) and three reads with reference sequences. Similarly, the other 9 samples also showed read profiles of between 1 and 10 bp contractions and expansions.

Eight variants occurred in target region 2, all intronic to *CADM1* (Fig. 2b). Var4 and Var8, both poly-T STRs, showed similar intra-sample read heterogeneity to Var3 for all samples, and were also only detected by Sniffles in Sample 2 and Sample 10 respectively, unable to be resolved, and classed as homozygous reference. The two indels, Var5 and Var7, both only had one non-reference allele, a 15 bp deletion and a 32 bp deletion respectively, and all samples were heterozygous. All three of the contiguous STRs, Var6, Var9, and Var11, were polymorphic. Var6 was again only detected by Sniffles but unable to be resolved and filtered out with classification of homozygous reference, however all 10 samples clearly showed insertions in their reads at the transition between the poly-A and poly-AG STRs. Both Var9 and Var11 had contraction and expansion alleles, with Var9 showing variance at the beginning of the poly-CT STR, and both variants at the transition between the two STRs. Var10 had polymorphic CA STR expansions.

Only one variant occurred in target region 3, Var12, intronic to *ASB3*, downstream of exon 2, and upstream to the 5ʹUTR of *CHAC2* (Fig. 2c). Var12 was a deletion of one repeat in reference sequence (TCTTTGGTGCTCCATTGATAAGAGCACC)_2_. Two variants occurred in target region 4, intronic to *MGMT*; an ~ 49–50 bp expansion internal to LINE-1 retrotransposon L1PA16 (Var13), and a poly-AT/T STR (Var14; Fig. 2d). Var14 was polymorphic, with both contraction and expansion alleles, and variability with which end of the repeat the variation occurred. Only one variant (Var15) occurred in target region 5, the last target region containing coding genes, with polymorphic contractions of a homopolymer T STR in the 3ʹUTR of *FOXP1* (Fig. 2e).

The final three target regions were all non-genic and had five total prioritised variants. Target region 6 contained one variant, an intergenic poly-A STR (Var16) intronic to non-coding element *LINC001787* (Fig. 2f). Var16 also had both contraction and expansion alleles. Three variants occurred in target region 7, a poly-AT STR (Var17), and two poly-AC STRs (Var18 and Var19). Var17 and Var18 were upstream of the long intergenic non-coding element, *LINC02228*, while Var19 was intronic to it (Fig. 2g). All three variants were polymorphic, with contraction and expansion alleles. Var17 was bisected by three contiguous A nucleotides in the reference sequence, and the variation in the alternate alleles detected in our samples all occurred at this point in the repeat. Finally, only one variant was reported for target region 8, an imperfect TCTT STR (Var20), intronic to *LINC00879* (Fig. 2h). Var20 had notable read heterogeneity, with reads showing multiple contractions and expansions across the length of the repeat.

### Prioritised SV annotations

All 20 prioritised variants also had predicted functional annotations within +/-2.5 kb in the UCSC genome browser tracks (Supplementary Table 11). The most common regulatory annotations were enhancers/enhancer-like-elements, and TFBS (Supplementary Table 11). For enhancer annotations, distal enhancer-like-elements, occurring in 60% of variant windows, were more frequent than proximal enhancer-like-elements, for which only Var12 had annotations. CCCTC-binding factor (CTCF) annotations were also not uncommon, occurring in nearly half of the variant windows. Expression quantitative trait loci (eQTLs) from GTEx brain tissues were reported in proximity to Var1 for *IP6K2*, Var2 for *IP6K2* and *NCKIPSD*, and Var3 for *SHISA5* (Supplementary Tables 11 and 12). The eQTLs near Var13 and 14 were both for *MGMT* (Supplementary Tables 11 and 12).

Var12 and Var15 were the only two variants that were in proximity to transcription start site annotations, with FANTOM5 robust transcription start site DPI peaks for *CHAC2* and *FOXP1*, respectively. Promotor annotations also only occurred in the vicinity of Var12, which had a promotor like signature (EH38E1998167), GeneHancer promotor elite (GH02J053766), and EDPnew promotor (Chac2_1) annotations. Finally, Var15 was the only variant overlapping micro-RNA (miRNA) binding sites.

We also searched for any GWAS catalogue hits [33] and identified 19 different hits occurring in the +/-2.5 kb windows around nine of variants Var1, Var2, Var4, Var5, Var6, Var8, Var12, Var13, and Var14 (Supplementary Table 11). Notably, the second most significant ANGI GWAS risk SNP, rs6589488 on chromosome 11, was located ~ 2.26 kb downstream of Var6 (Supplementary Table 11). Phenotypes specifically associated with the other 18 overlapping GWAS hits included brain and psychometric traits, specifically superior temporal area (unadjusted for global measures), brain morphology (MOSTest), insomnia, household income (MTAG), cognitive aspects of educational attainment, morningness, and morning person; immune traits, specifically macrophage inflammatory protein 1b levels and eosinophil counts; metabolic traits, specifically HDL cholesterol, thioredoxin domain-containing protein 12 levels, response to cytidine analogues (cytosine arabinoside), mean corpuscular volume, mean corpuscular haemoglobin, and estimated glomerular filtration rate (creatinine); anthropometric traits, specifically waist circumference adjusted for body mass index (BMI), height, whole body fat mass (UKB data field 23100), weight, BMI, childhood BMI, South Asian ancestry bone mineral density; and finally the congenital disorder orofacial clefts.

## Discussion

The primary focus of this pilot study was to perform a detailed characterisation of potentially polymorphic structural variation that may contribute to the heritability of these AN risk-associated regions. We successfully applied targeted nanopore sequencing and a bioinformatics pipeline to prioritise 20 SVs across the eight target regions of potential interest for investigation in larger association and functional studies (Table 3). The polymorphism of the variants between the 10 samples and the frequent intra-sample variability between calling algorithms observed here (Table 3; Supplementary Tables 5 and 6) potentially indicate poor representation in the reference genome and reflect the findings from current genome assembly studies [12]. All 20 variants also showed at least one non-reference allele in samples from the HPRC track in the UCSC browser, however allele distributions varied compared with our cohort, particularly as genotype consensus was so challenging (Supplementary Table 7). For example, we point to Var3, an A homopolymer STR, occurring at the tail of an AluY element and intergenic to *LINC02585* (Fig. 2a). The heterogeneity within the reads of each of the samples in this study reflect difficulty aligning to the reference genome. Concomitantly the UCSC Genome Browser shows poor mappability arguing against the accuracy of the reference sequence for Var3 [34]. Thus, these regions may simply be absent from the current human reference genome, and the variation being detected is normal and the reference genome is incorrect, they are highly unstable regions prone to mutability, or the variability is due to consistent nanopore sequencing error, although the variation in the HPRC samples would argue against this. It is worthwhile noting that the HPRC assembly genomes are a largely non-European cohort, which may affect allele frequencies. Regardless, it is imperative that such areas of the genome be further queried to help understand the full spectrum of genetic variation in human health and disease.

Sequence instability promoting mutability is increasingly being highlighted with the functional impacts of TRs. It is important to continue to identify the specific nature of length and composition differences of these regions as the mutability characteristic of repetitive elements disposes them to variable penetrance. Variable penetrance may help explain the disorder heterogeneity apparent in AN, as well as identifying subthreshold traits [35]. Overall, 15 of our prioritised variants were STRs, and all but one had regulatory element annotations, especially TFBS and enhancers, within 2.5+\- kb windows, indicating plausible regulatory influences. Despite not directly overlapping these regulatory elements, expression STRs have been shown to have effects when located within 100 kb of the transcription start site, promotors, and enhancers, with the significance of impact increasing as distance to affected element decreases [4]. Moreover, STRs have been shown elsewhere to affect gene expression cumulatively and indirectly by altering the binding affinity of neighbouring TFBS for TFs [36]. For example, the overlapping of TFBS for TFs for *CADM1* and *FOXP1* is suggestive evidence in favour of variants Var4, Var8 and Var10, and Var15, respectively. Finally, the difficulty in characterising variants with annotation tools in targets 6–8 could potentially be a result of these being understudied non-coding regions. These three regions appear to fall in gene deserts, with nearest coding genes being kilobases away.

We direct specific attention to variants Var2, Var12, and Var15, and speculate about several possible functional implications at the molecular level in AN pathobiology. First, we highlight Var15, a poly T contraction in the 3ʹUTR of *FOXP1*. The constraint metric reported in gnomAD browser for *FOXP1* indicates that the gene has a low variant tolerance [37]. The 3ʹUTR of genes is important in post-transcriptional mRNA stability and processing, particularly through miRNA binding. Var15 occurs in proximity to five different possible miRNA binding sites (Supplementary Table 11), indicating a potential for it to disrupt these areas [38]. STR variations in the *FOXP1* 3ʹUTR have previously been implicated in cases of autism spectrum disorder (ASD) [39]. ASD and autistic features have reported phenotypic similarities and genetic correlation with AN [40]. We posit that the contraction of the T homopolymer could potentially influence *FOXP1* expression at the post-transcriptional processing stage and warrants further investigation [38].

Var2 is a variable SVA-D element, a subtype of the hominid specific SVA elements. SVA elements are still relatively evolutionarily young and active compared to other retrotransposons and have previously been identified as hotspots for variation and recombination [41, 42]. Moreover, and a potentially intriguing functional role of the variant in AN biology, SVAs have been implicated as neuropeptide expression moderators [42]. The genes surrounding Var2 (*IP6K2*, *NCKIPSD*, *PRKAR2A*), which have previously been implicated in AN, have roles in cell maintenance and immune system regulation [43, 44]. Also, *SHISA5*, which occurred just proximal to target region 1 and had a similar SVA-D variation as Var2, was significantly associated with downregulation of expression in AN TWAS analysis of cerebellum tissue [44]. SVAs are also known to be enriched in genic regions [45], such as target region 1. By influencing expression mechanisms of surrounding genes, Var2 may impact development of neuronal functions, which could be of relevance to the aetiology of AN and further exploration of SVA elements in AN biology would be interesting.

Var12 overlaps both *ASB3* and *CHAC2*, genes significantly associated via gene-wise analysis for AN [2]. Var12 could potentially affect expression of *ASB3* and *CHAC2* pleiotropically, disrupting several TFBS for both genes, as well as promotors and proximal enhancers annotated for *CHAC2*. Functionally, *CHAC2* encodes the ChaC2 enzyme, which is a critical player in glutathione redox metabolism, and *ASB3* has been implicated in tumour necrosis factor alpha related ubiquitination and proteome degradation [46, 47]. In light of these functions, Var12 potential impacts on expression could be broadly relevant to metabolic cellular processing in AN pathobiology and disorder maintenance [2, 43]. The variants detected and prioritised in this study have intriguing putative implications in AN pathobiology but should be interpreted cautiously and require further investigation.

### Limitations

This was an exploratory study designed to evaluate the hypothesis that poorly resolved and understudied structural variants may reside in AN GWAS regions and contribute to significant associations via LD. There are several limitations inherent to this study design. First, although we examined samples that contributed to a large GWAS on AN, it was a small (*n* = 10) sample without controls or sufficient statistical power to draw inferences about association with AN. Second, the bioinformatics tools and databases used for genotyping and annotation are a bottleneck for data analysis, each with inherent limitations and biases. Databases and reference genome material are limited by the technologies used for their construction and are thus unable to accurately inform the full spectrum of human genomic variation. The potential implications of the prioritised variants, particularly Var2, Var12, and Var15, are speculative, and should be interpreted with caution. Our data reaffirm the current push to improve poorly annotated regions of the genome, as these may be sites of variation functionally relevant to AN [11]. It is also important to note that while we focussed on the 100 kb immediately either side of each of the eight lead SNPs, it is possible that LD blocks extend outside of these 200 kb windows, and it would be valuable to increase the search area in future studies with increased power for detection. Finally, nanopore sequencing error may have artificially inflated the number of variants detected, as well as the observed heterogeneity between samples.

## Conclusion

Our proof-of-principle study highlights strengths and limitations of targeted nanopore sequencing in capturing the spectrum of genetic variation in surrounding regions of loci identified by AN GWAS. All 20 prioritised variants were poorly represented in the GRCh38 reference genome. Although speculative, variants such as the polymorphic SVA-D and *FOXP1* 3ʹUTR poly-T STR could contribute to mechanisms of phenotypic risk through regulation of several neighbouring genes implicated in AN biology. We emphasise the need to characterise repetitive and transposable elements and understand their influence on expression mechanisms of genes biologically implicated in AN. The approach developed here could be applied to streamline investigations of additional genomic regions with the release of larger ED data sets. Identifying functional genetic architecture underpinning this devastating disorder could be an important path to informing development of personalised therapeutics and treatment.

## Electronic supplementary material

Below is the link to the electronic supplementary material.

Supplementary Material 1

Supplementary Material 2

Supplementary Material 3

Supplementary Material 4

## Data Availability

The anorexia nervosa GWAS summary statistics are publicly available at https://doi.org/10.6084/m9.figshare.14671980. The UCSC genome browser is publicly available at https://genome.ucsc.edu/cgi-bin/hgGateway. Variant calls, linkage disequilibrium graphs and targeted nanopore sequencing statistics produced in this study can be found in the Results section and supplementary files. The raw sequencing data produced in this study has local ethics restrictions on deposition in public repositories and are available on reasonable request. Please contact Natasha Berthold at natasha.berthold@research.edu.au and Martin Kennedy at martin.kennedy@otago.ac.nz.

## Abbreviations

AN: Anorexia nervosa
ASD: Autism spectrum disorder
BMI: Body mass index
CTCF: CCCTC-binding factor
ED: Eating disorder
GWAS: Genome wide association study
IGV: Integrative Genomics Viewer
Indel: Insertion/deletion
miRNA: Micro-RNA
ONT: Oxford Nanopore Technologies
SNPs: Single nucleotide polymorphisms
STR: Short tandem repeat
SV: Structural variant
SVA-D: Subfamily D of the sine-VNTR-Alu like elements
TFBS: Transcription factor binding site
TR: Tandem repeat
UTR: Untranslated region
VEP: Variant effect predictor
