# Supplementary material for: Nanopore sequencing as a novel method of characterising anorexia nervosa risk loci
Source: BMC Genomics. 2024 Dec 31;25:1262. doi: 10.1186/s12864-024-11172-7 (PMC11687000; doi:10.1186/s12864-024-11172-7)
Supplement: Supplementary file 1 — Supplementary Material 1 [file 12864_2024_11172_MOESM1_ESM.pdf]

Supplementary Figure 1. Coverage statistics from nanopore sequencing showing the average number of reads per base in the first panel (i.e. the coverage) and the fraction of total reads that was equal to or greater than the coverage. This was done for each sample for both off-target areas (upper two panels) and on-target areas (lower two panels). **a,b)** Sample 1 **c,d)** Sample 2, **e,f)** Sample 3 **g,h)** Sample 4 **i,j)** Sample 5 **k,l)** Sample 6 **m,n)** Sample 7 **o,p)** Sample 8 **q,r)** Sample 9 **s,t)** Sample 10

1a.

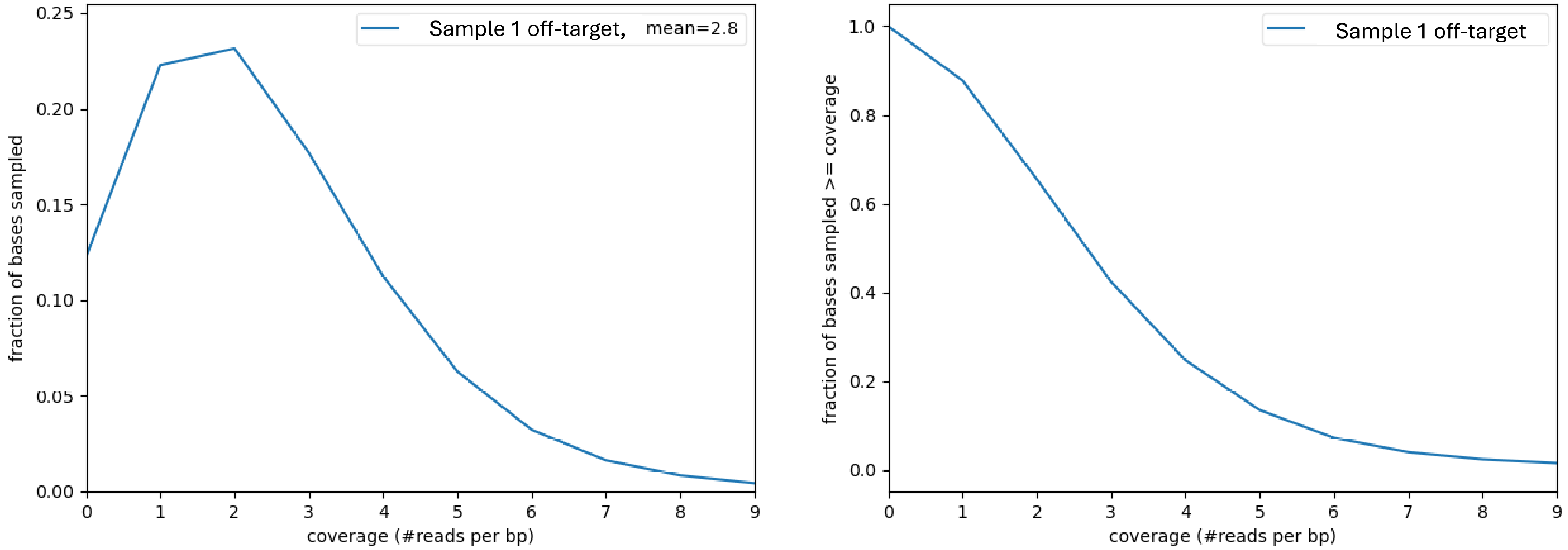

Sample 1 off-target, mean=2.8
fraction of bases sampled
coverage (#reads per bp)
Sample 1 off-target
fraction of bases sampled >= coverage
coverage (#reads per bp)

1b.

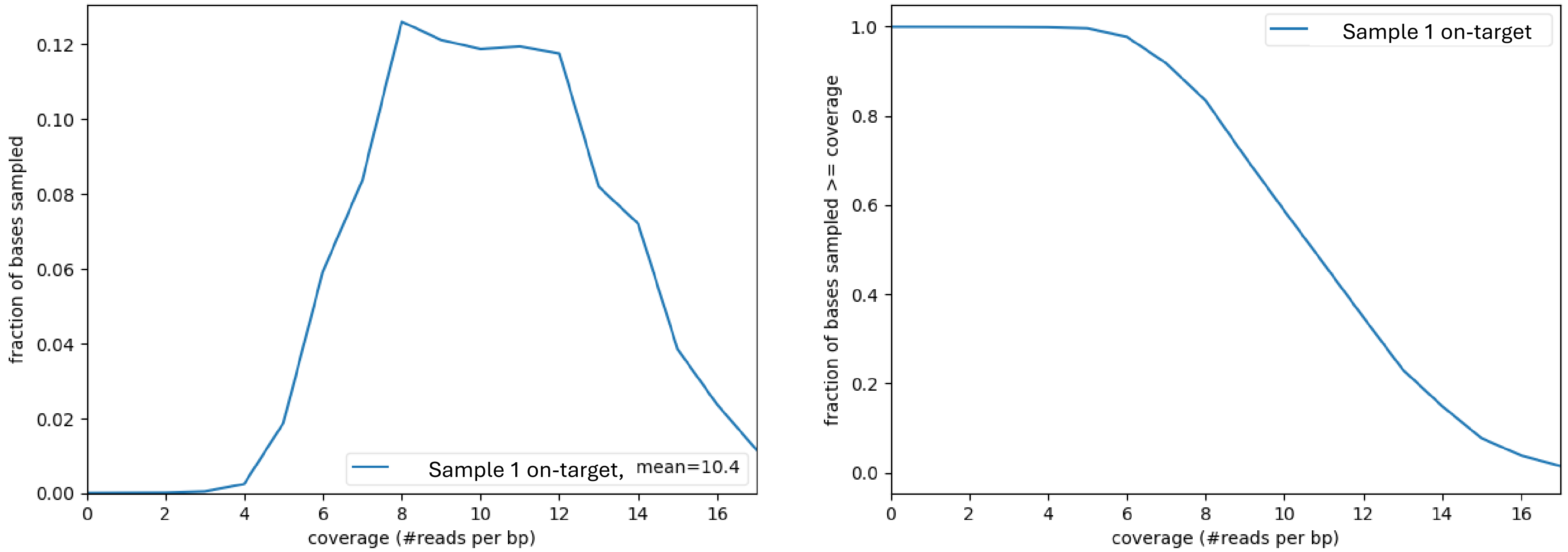

Sample 1 on-target, mean=10.4
fraction of bases sampled
coverage (#reads per bp)
Sample 1 on-target
fraction of bases sampled >= coverage
coverage (#reads per bp)

1c.

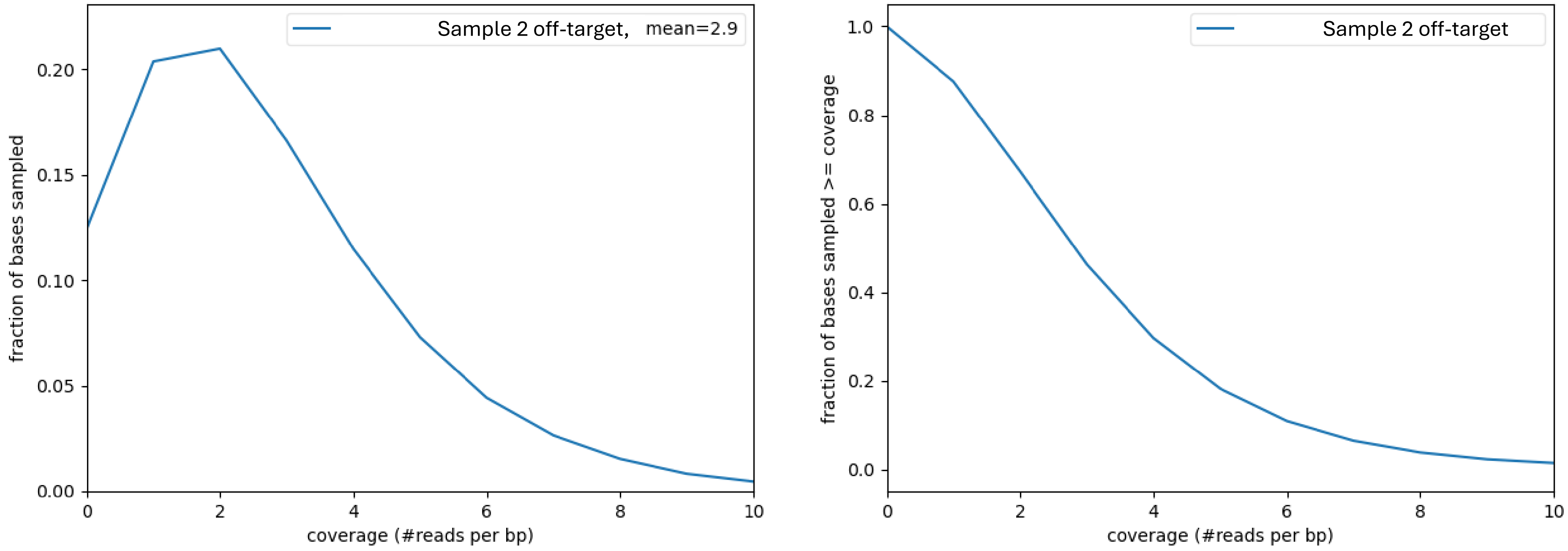

Sample 2 off-target, mean=2.9
fraction of bases sampled
coverage (#reads per bp)
Sample 2 off-target
fraction of bases sampled >= coverage
coverage (#reads per bp)

1d.

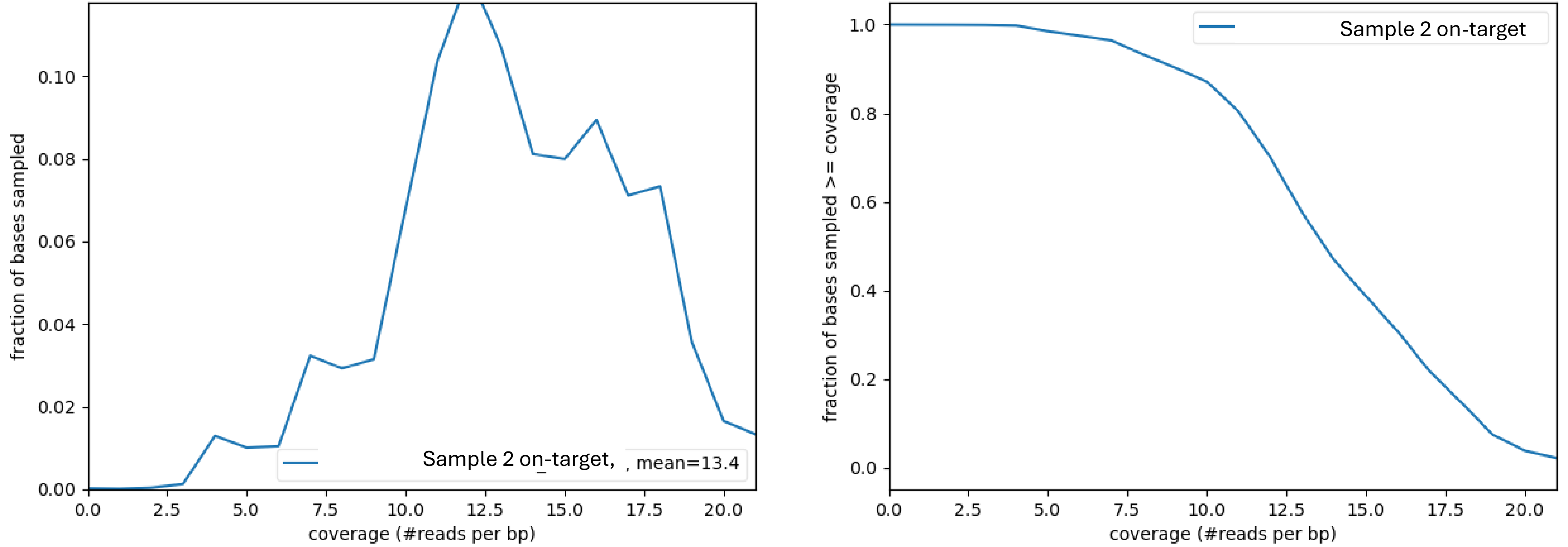

Sample 2 on-target, , mean=13.4
fraction of bases sampled
coverage (#reads per bp)
Sample 2 on-target
fraction of bases sampled >= coverage
coverage (#reads per bp)

1e.

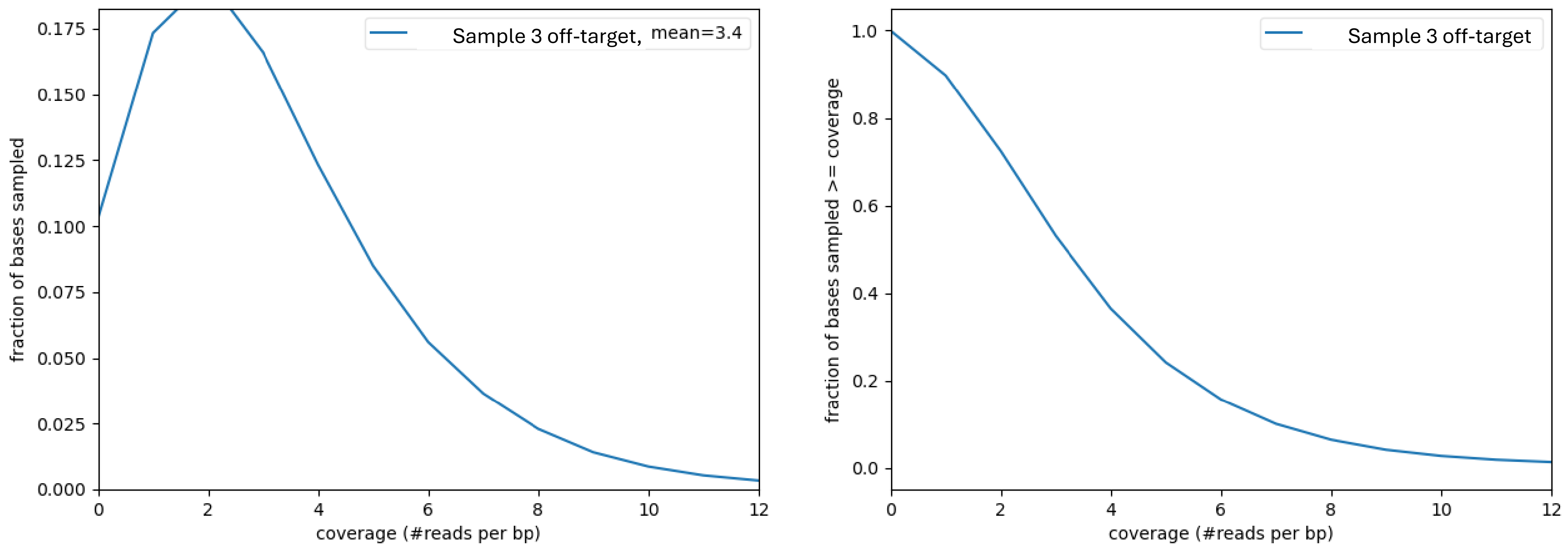

Sample 3 off-target, mean=3.4
fraction of bases sampled
coverage (#reads per bp)
Sample 3 off-target
fraction of bases sampled >= coverage
coverage (#reads per bp)

1f.

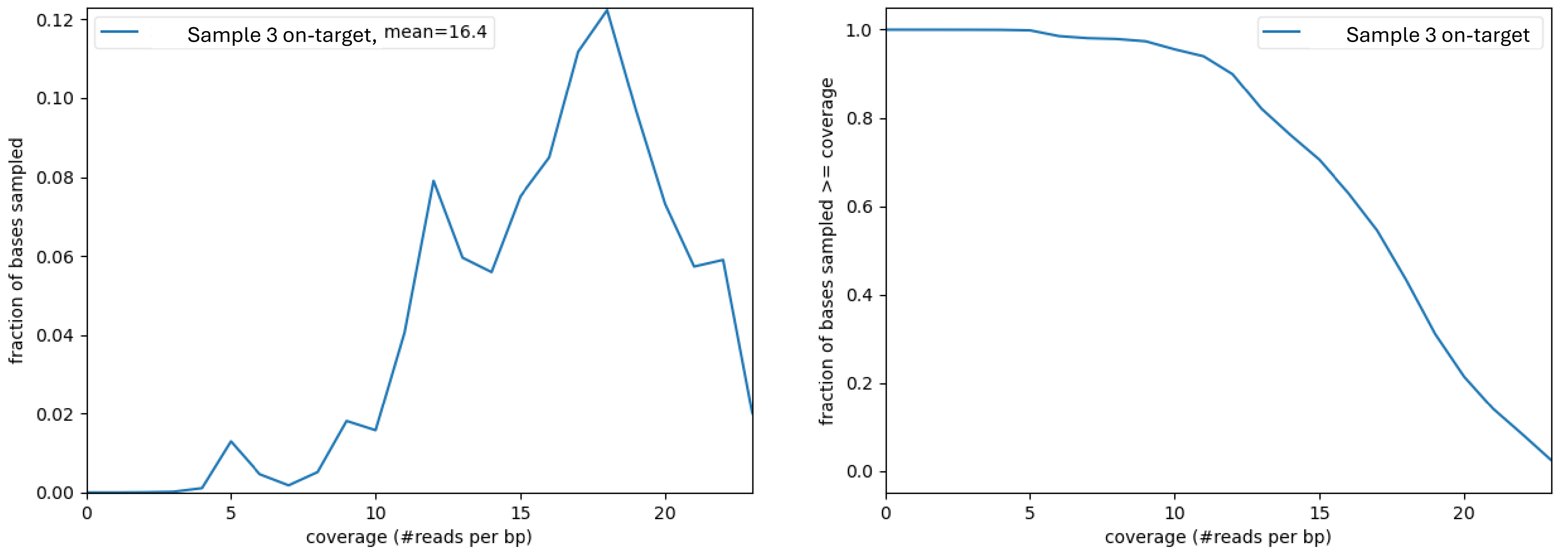

Sample 3 on-target, mean=16.4
fraction of bases sampled
coverage (#reads per bp)
Sample 3 on-target
fraction of bases sampled >= coverage
coverage (#reads per bp)

1g.

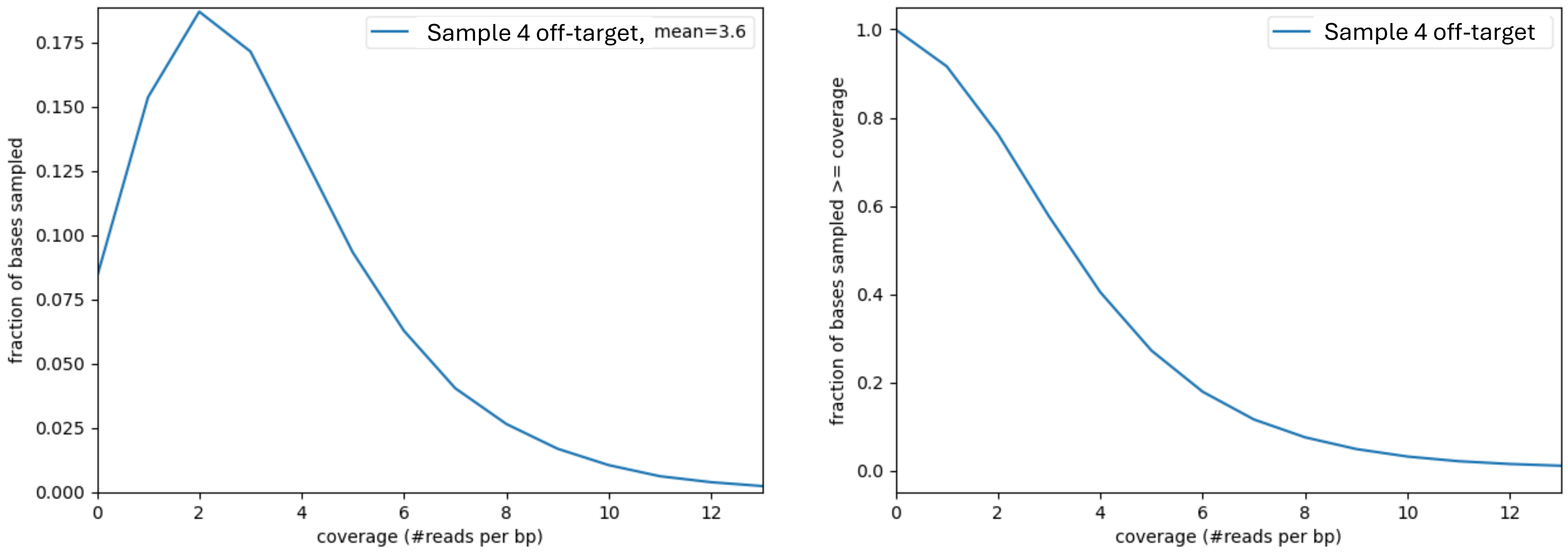
Sample 4 off-target, mean=3.6
fraction of bases sampled
coverage (#reads per bp)
Sample 4 off-target
fraction of bases sampled >= coverage
coverage (#reads per bp)

1h.

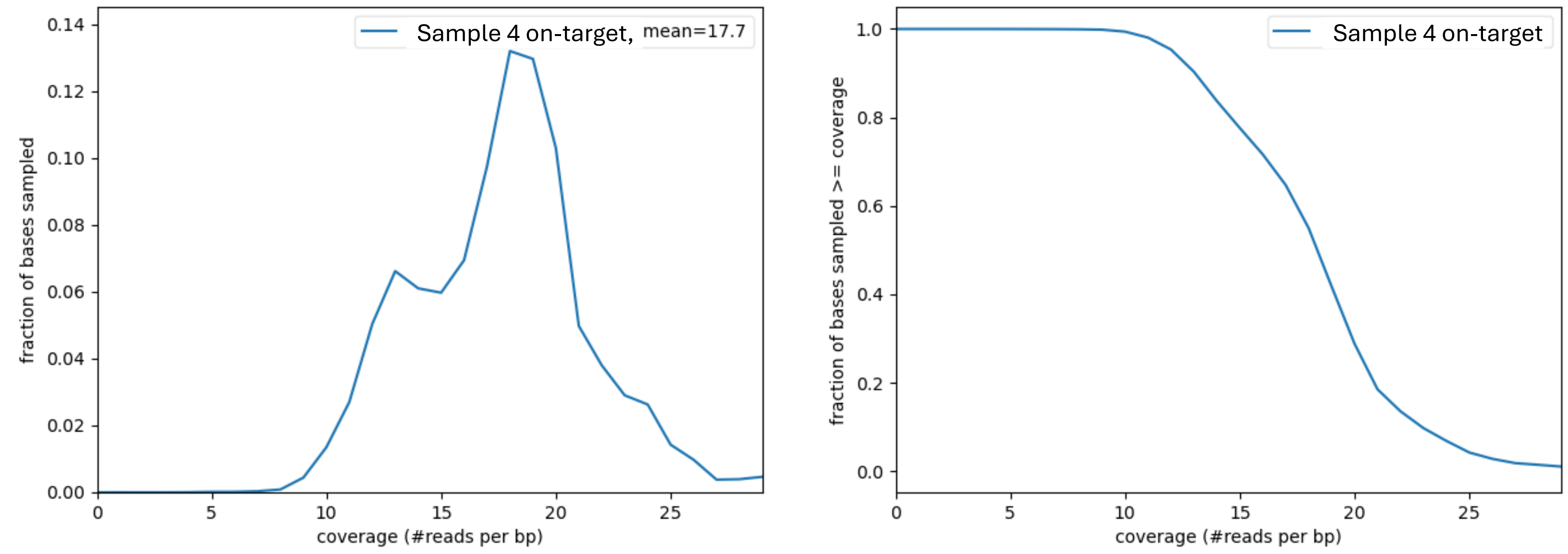
Sample 4 on-target, mean=17.7
fraction of bases sampled
coverage (#reads per bp)
Sample 4 on-target
fraction of bases sampled >= coverage
coverage (#reads per bp)

1i.

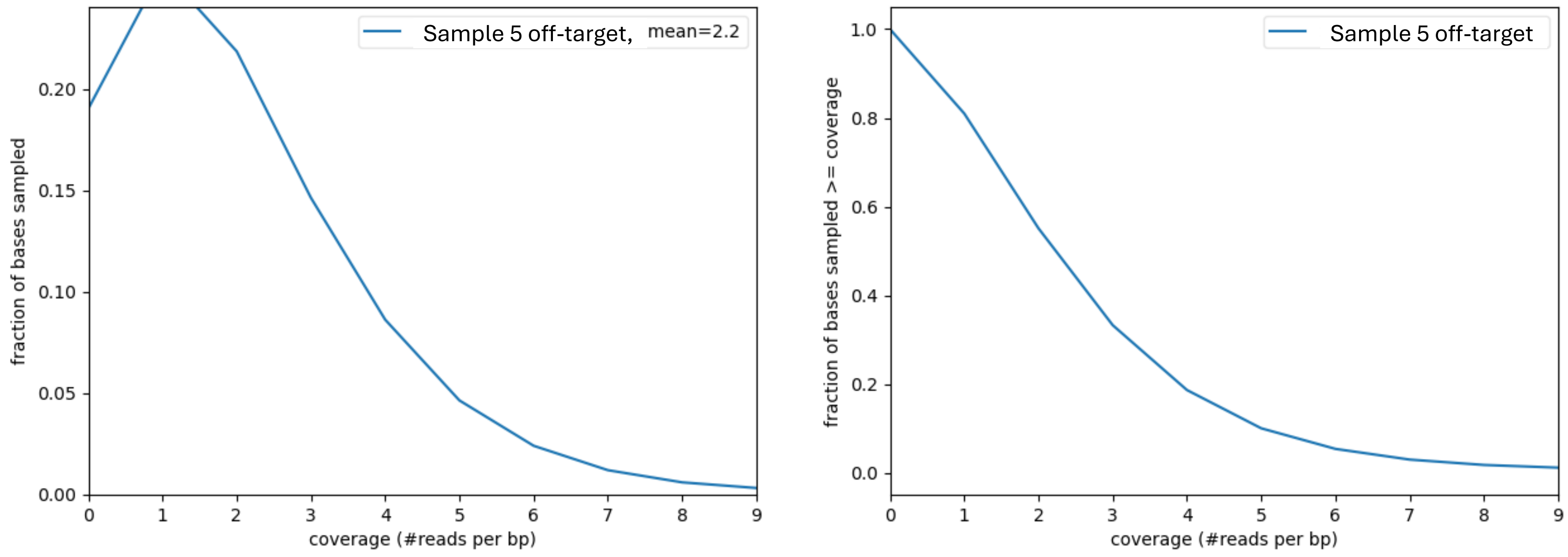
Sample 5 off-target, mean=2.2
fraction of bases sampled
coverage (#reads per bp)
Sample 5 off-target
fraction of bases sampled >= coverage
coverage (#reads per bp)

1j.

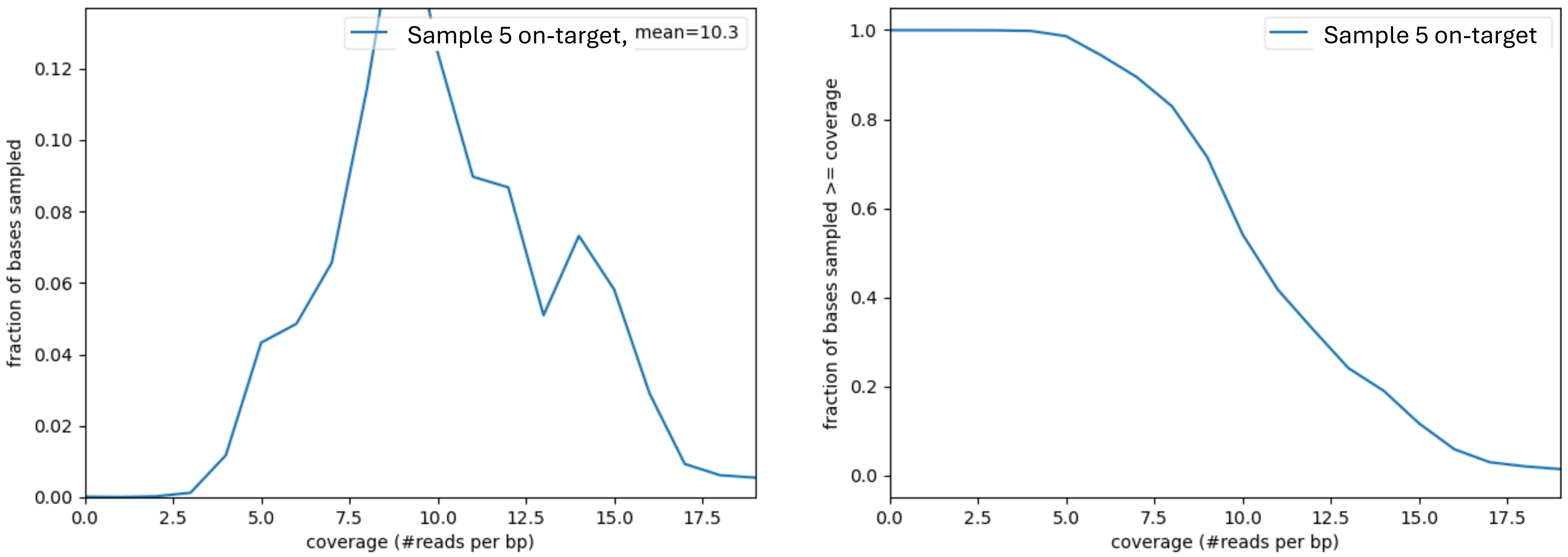
Sample 5 on-target, mean=10.3
fraction of bases sampled
coverage (#reads per bp)
Sample 5 on-target
fraction of bases sampled >= coverage
coverage (#reads per bp)

1k.

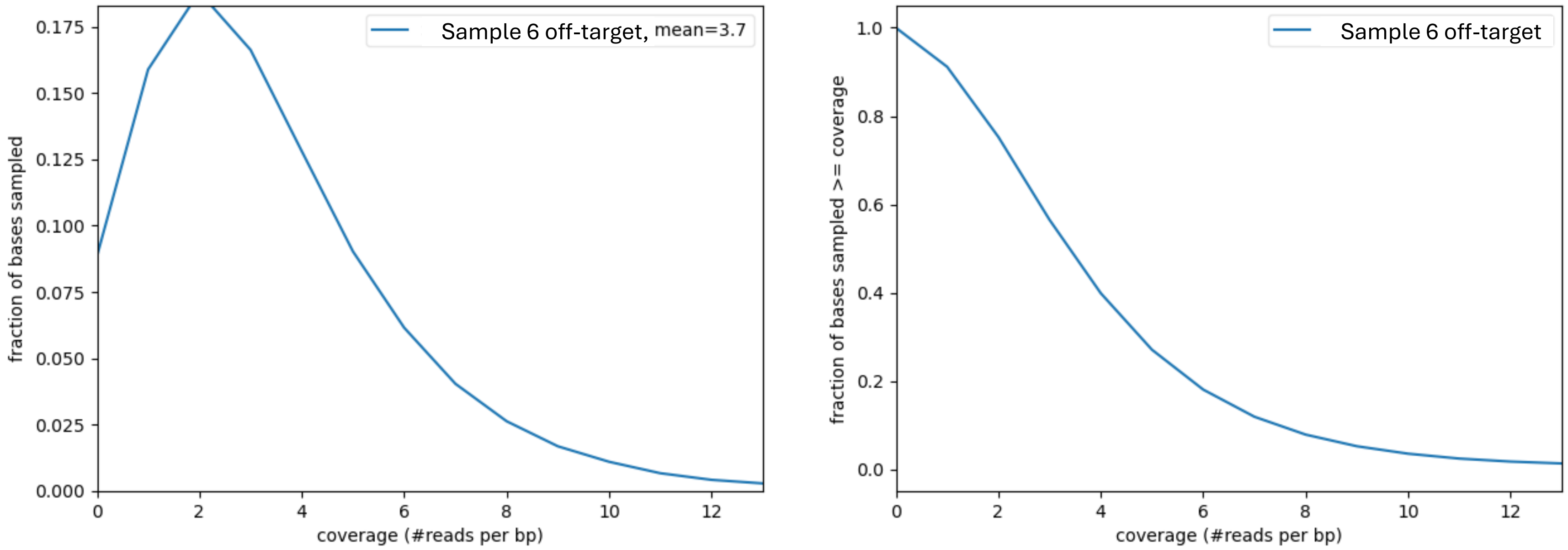
Sample 6 off-target, mean=3.7
fraction of bases sampled
0.175
0.150
0.125
0.100
0.075
0.050
0.025
0.000
0
2
4
6
8
10
12
coverage (#reads per bp)
Sample 6 off-target
fraction of bases sampled >= coverage
1.0
0.8
0.6
0.4
0.2
0.0
coverage (#reads per bp)

1l.

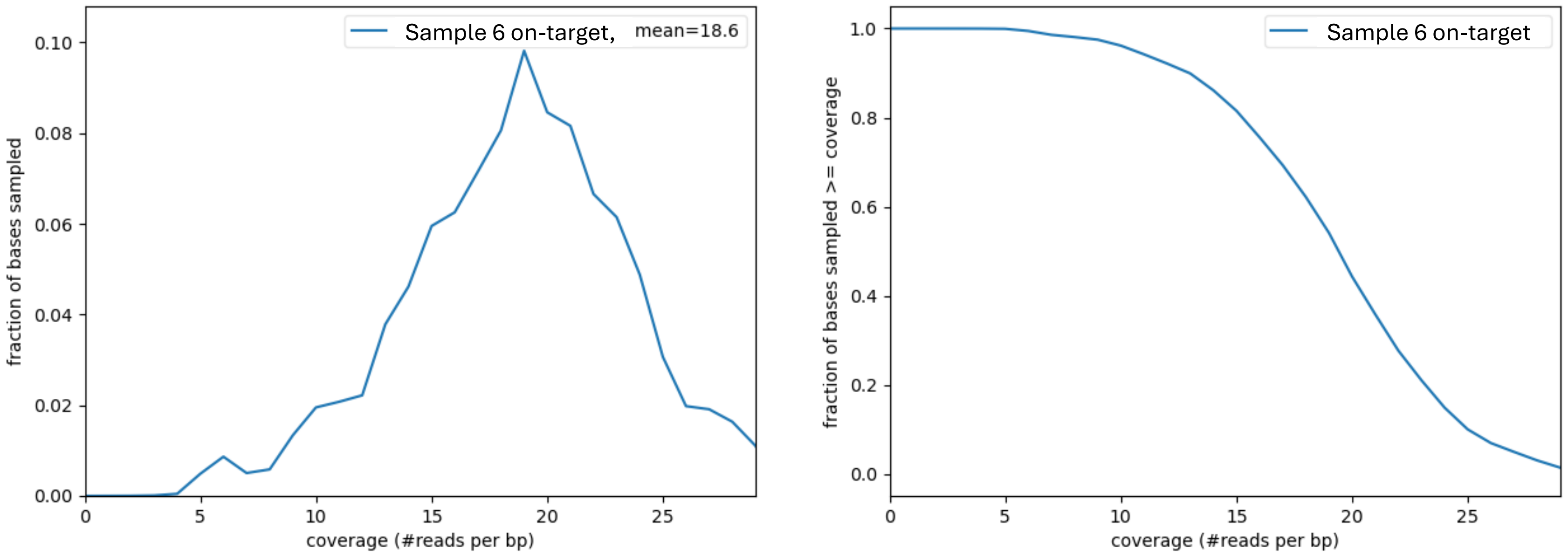
Sample 6 on-target, mean=18.6
fraction of bases sampled
0.10
0.08
0.06
0.04
0.02
0.00
0
5
10
15
20
25
coverage (#reads per bp)
Sample 6 on-target
fraction of bases sampled >= coverage
1.0
0.8
0.6
0.4
0.2
0.0
coverage (#reads per bp)

1m.

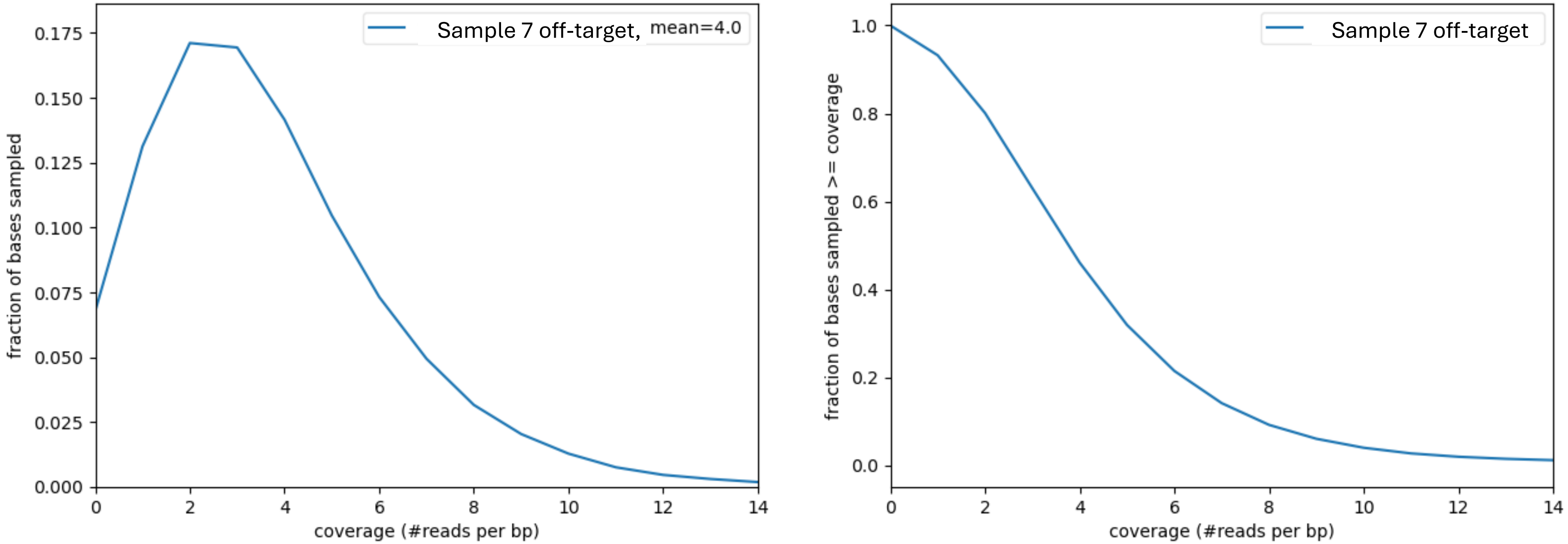

Sample 7 off-target, mean=4.0
fraction of bases sampled
coverage (#reads per bp)
Sample 7 off-target
fraction of bases sampled >= coverage
coverage (#reads per bp)

1n.

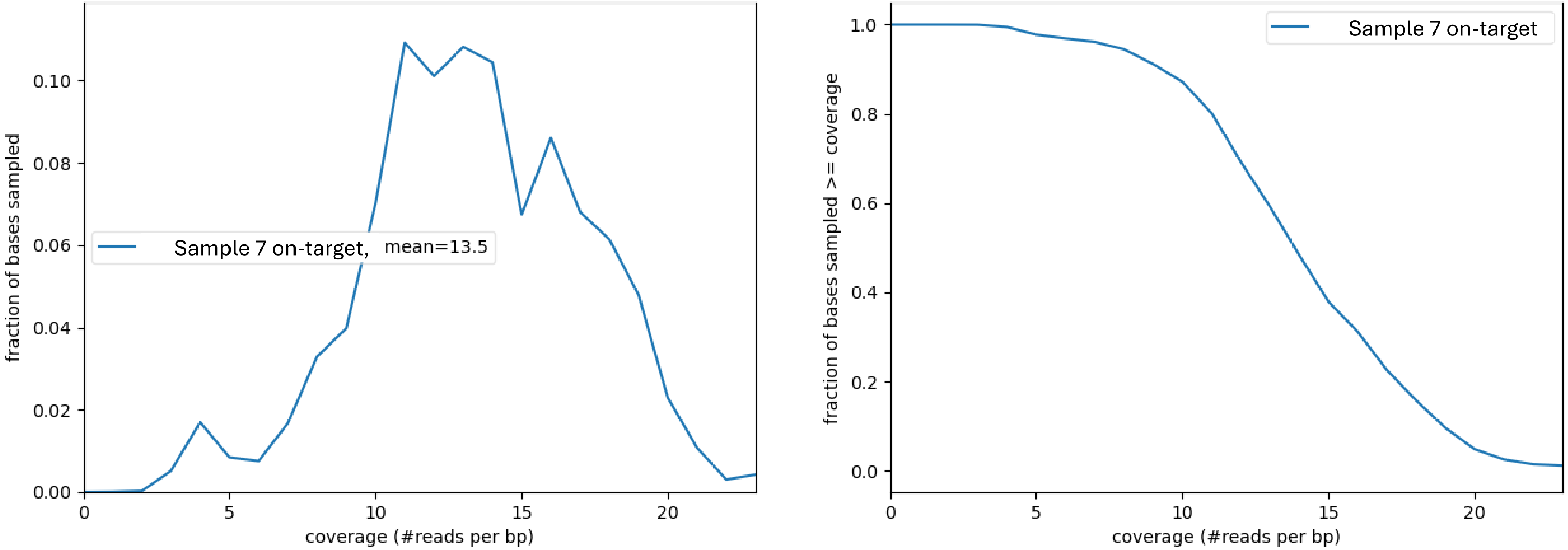

Sample 7 on-target, mean=13.5
fraction of bases sampled
coverage (#reads per bp)
Sample 7 on-target
fraction of bases sampled >= coverage
coverage (#reads per bp)

1o.

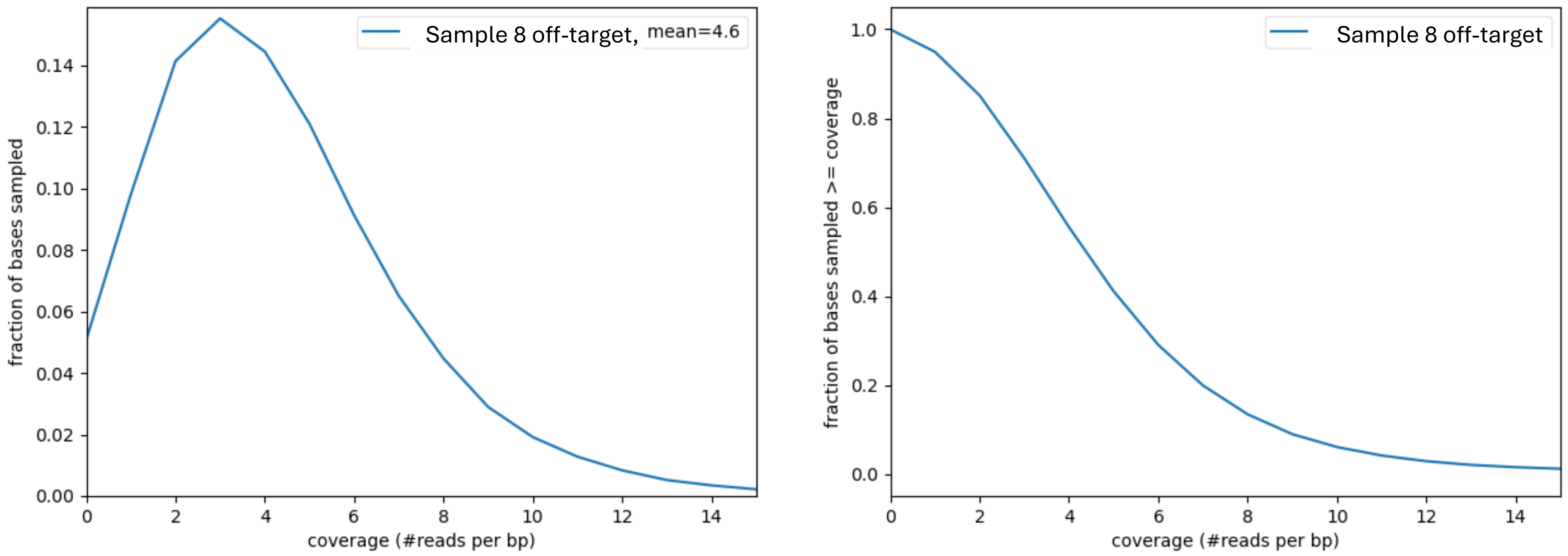
Sample 8 off-target, mean=4.6
fraction of bases sampled
coverage (#reads per bp)
0.00
0.02
0.04
0.06
0.08
0.10
0.12
0.14
0
2
4
6
8
10
12
14
Sample 8 off-target
fraction of bases sampled >= coverage
0.0
0.2
0.4
0.6
0.8
1.0

1p.

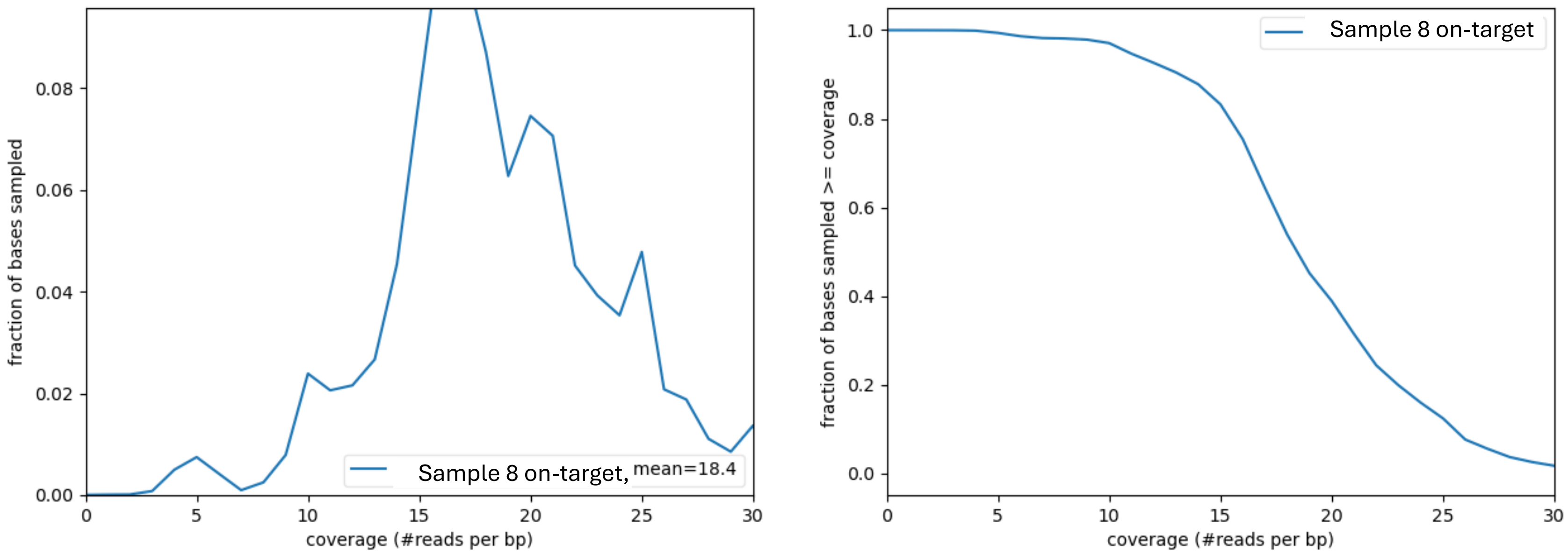
Sample 8 on-target, mean=18.4
fraction of bases sampled
coverage (#reads per bp)
0.00
0.02
0.04
0.06
0.08
0
5
10
15
20
25
30
Sample 8 on-target
fraction of bases sampled >= coverage
0.0
0.2
0.4
0.6
0.8
1.0

1q.

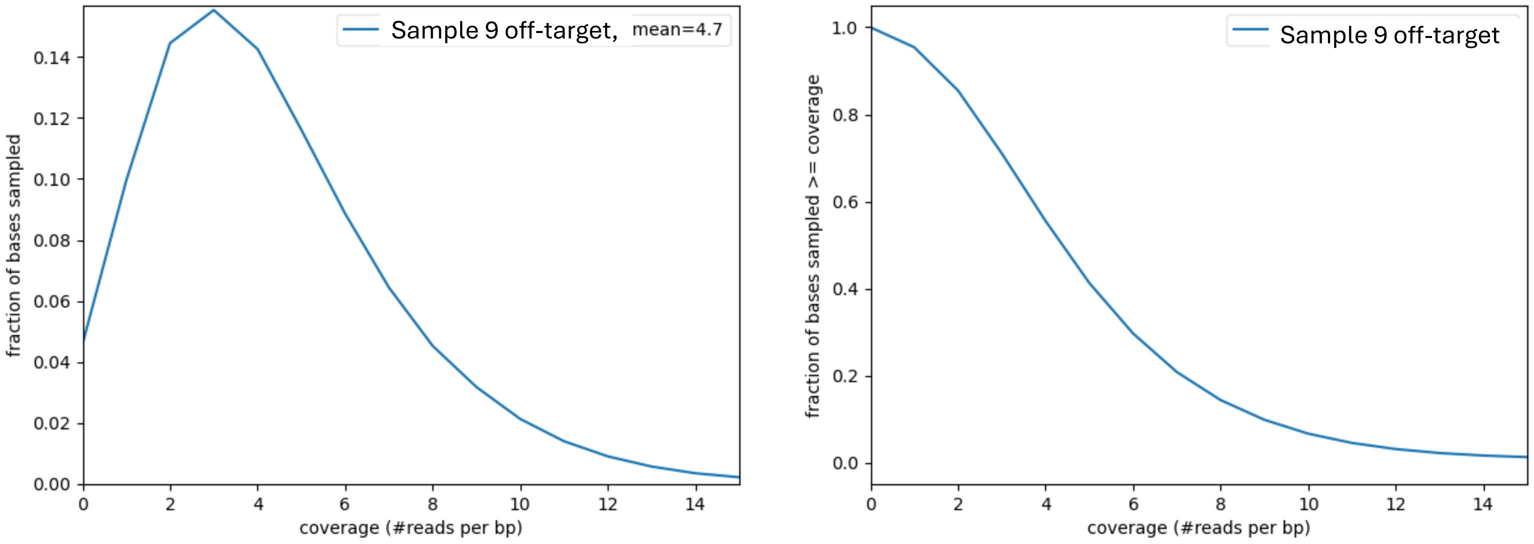
Sample 9 off-target, mean=4.7
fraction of bases sampled
coverage (#reads per bp)
Sample 9 off-target
fraction of bases sampled >= coverage
coverage (#reads per bp)

1r.

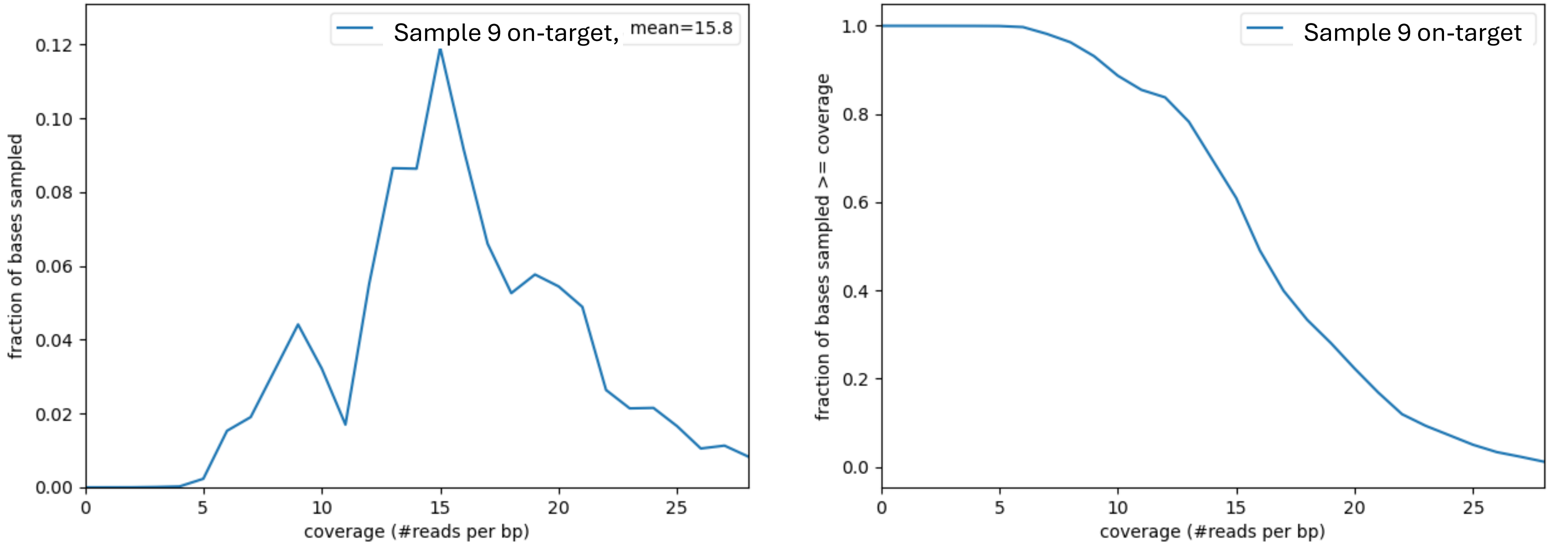
Sample 9 on-target, mean=15.8
fraction of bases sampled
coverage (#reads per bp)
Sample 9 on-target
fraction of bases sampled >= coverage
coverage (#reads per bp)

1s.

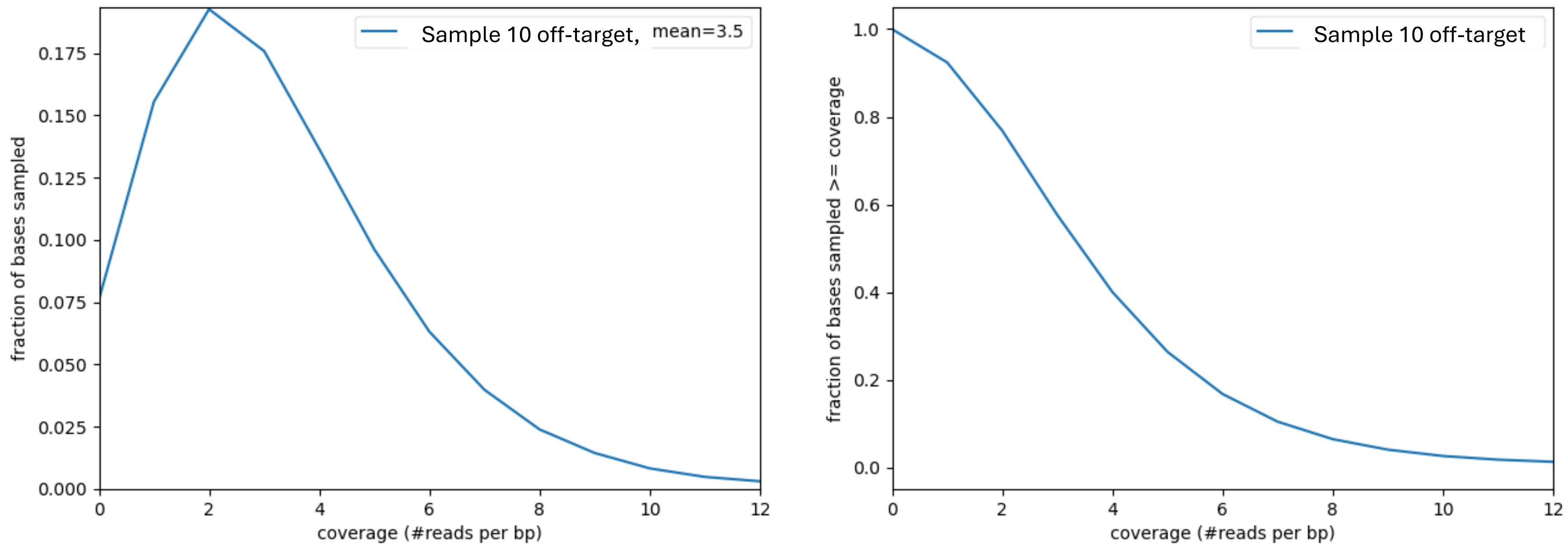

Sample 10 off-target, mean=3.5
fraction of bases sampled
0.000
0.025
0.050
0.075
0.100
0.125
0.150
0.175
0
2
4
6
8
10
12
coverage (#reads per bp)
Sample 10 off-target
fraction of bases sampled >= coverage
0.0
0.2
0.4
0.6
0.8
1.0
0
2
4
6
8
10
12
coverage (#reads per bp)

1t.

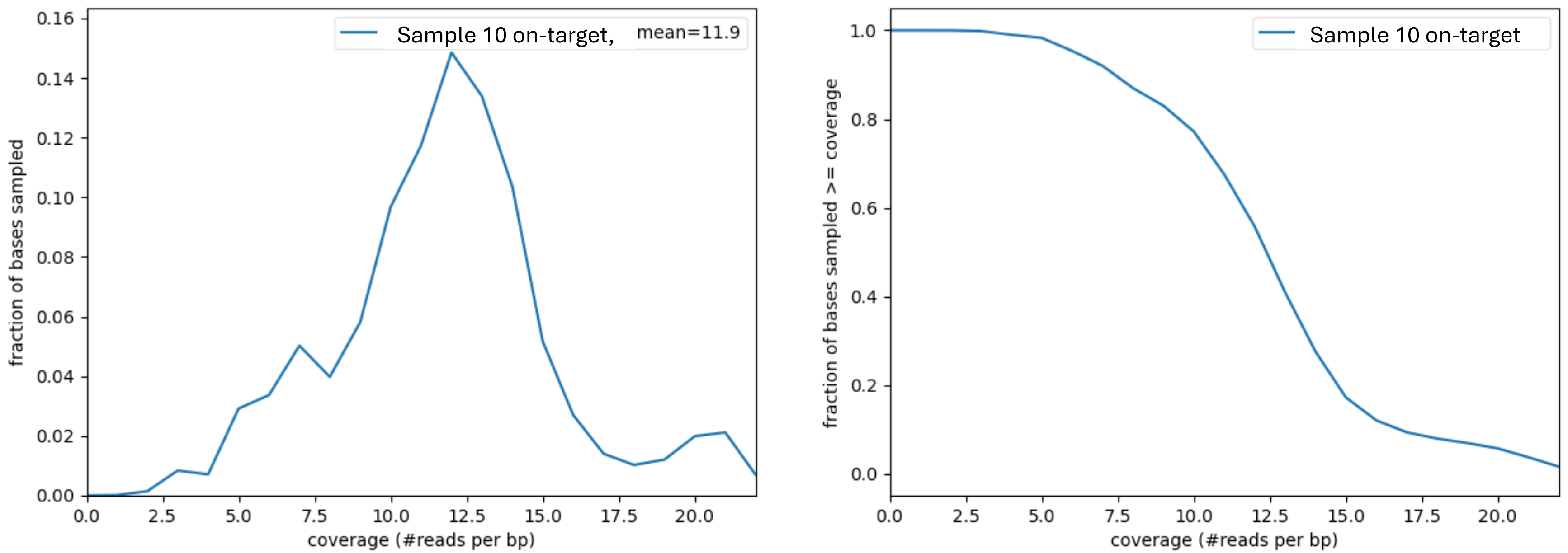

Sample 10 on-target, mean=11.9
fraction of bases sampled
0.00
0.02
0.04
0.06
0.08
0.10
0.12
0.14
0.16
0.0
2.5
5.0
7.5
10.0
12.5
15.0
17.5
20.0
coverage (#reads per bp)
Sample 10 on-target
fraction of bases sampled >= coverage
0.0
0.2
0.4
0.6
0.8
1.0
0.0
2.5
5.0
7.5
10.0
12.5
15.0
17.5
20.0
coverage (#reads per bp)
