# Supplementary material for: Nanopore sequencing as a novel method of characterising anorexia nervosa risk loci
Source: BMC Genomics. 2024 Dec 31;25:1262. doi: 10.1186/s12864-024-11172-7 (PMC11687000; doi:10.1186/s12864-024-11172-7)

Supplementary Figure 2a. Variant Effect Predictor annotation statistics of all variants from all variant callers, across all samples and all eight target regions. The table shows numeric statistics. The statistics for functional sequence consequences and coding consequences are depicted as percentage breakdown of the total variants in the two pie charts respectively. **2b-i.** Variant effect predictor annotation statistics for all variants from all variant callers and samples by target region from target region 1 to target region 8, respectively. Of note, there are no coding consequences reported for target regions 2 (2c), 6 (2g), 7 (2h) and 8 (2i).

2a. Total variant summary

Category	Count
Variants processed	18980
Variants filtered out	0
Novel / existing variants	469 (2.5) / 18511 (97.5)
Overlapped genes	33
Overlapped transcripts	297
Overlapped regulatory features	222

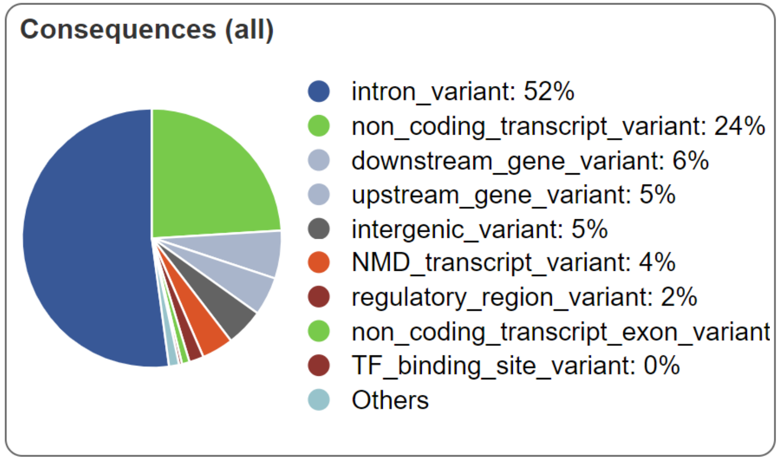

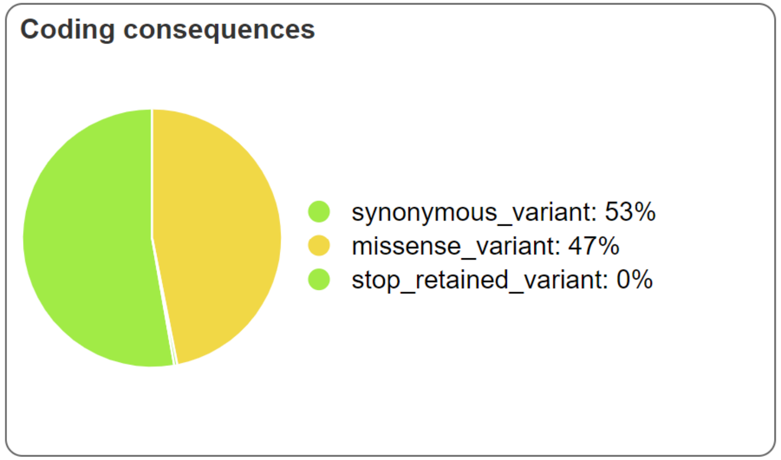

2b. Target region 1 variant summary

Category	Count
Variants processed	615
Variants filtered out	0
Novel / existing variants	33 (5.4) / 582 (94.6)
Overlapped genes	13
Overlapped transcripts	105
Overlapped regulatory features	38

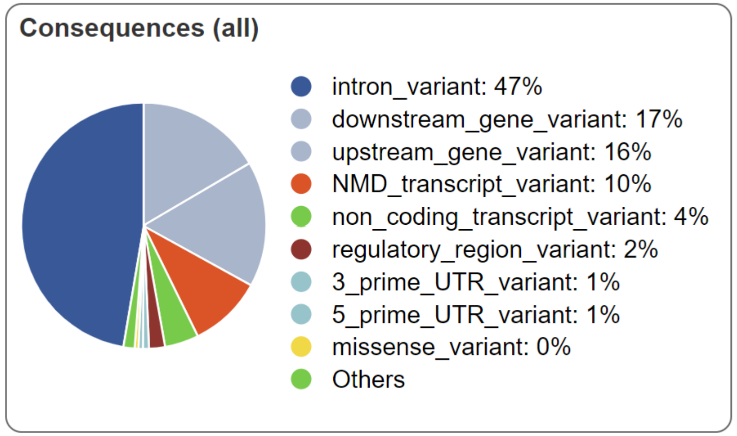

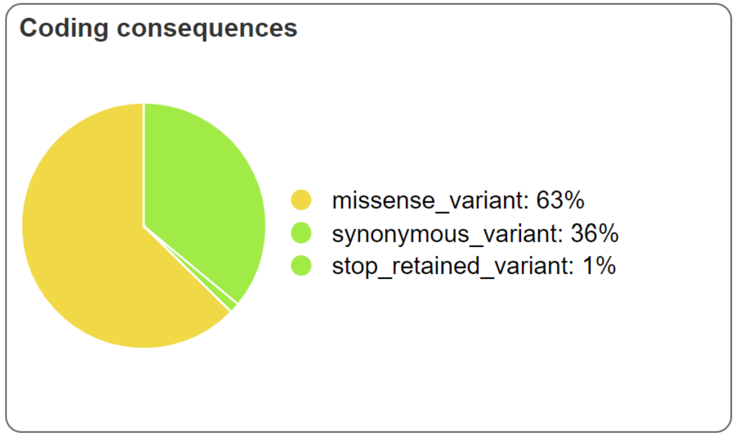

2c. Target region 2 variant summary

Category	Count
Variants processed	2268
Variants filtered out	0
Novel / existing variants	51 (2.2) / 2217 (97.8)
Overlapped genes	2
Overlapped transcripts	21
Overlapped regulatory features	42

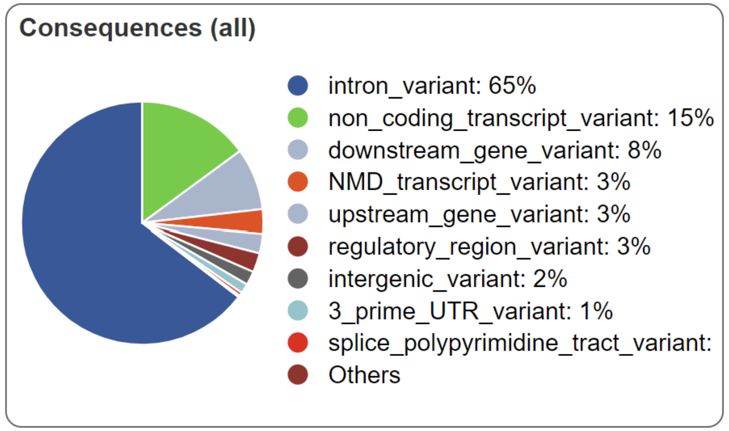

2d. Target region 3 variant summary

Category	Count
Variants processed	1875
Variants filtered out	0
Novel / existing variants	49 (2.6) / 1826 (97.4)
Overlapped genes	6
Overlapped transcripts	66
Overlapped regulatory features	25

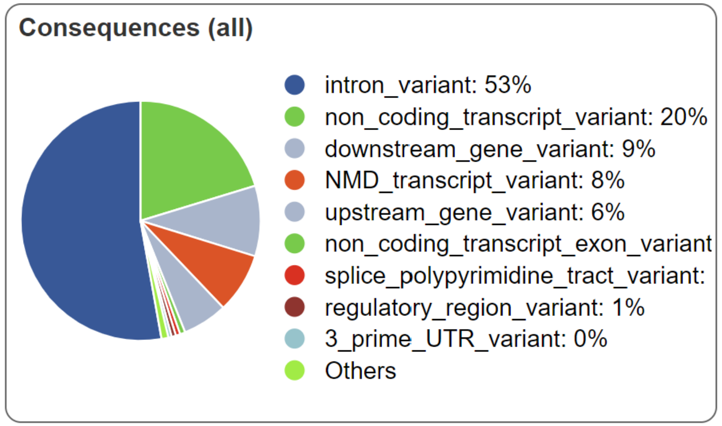

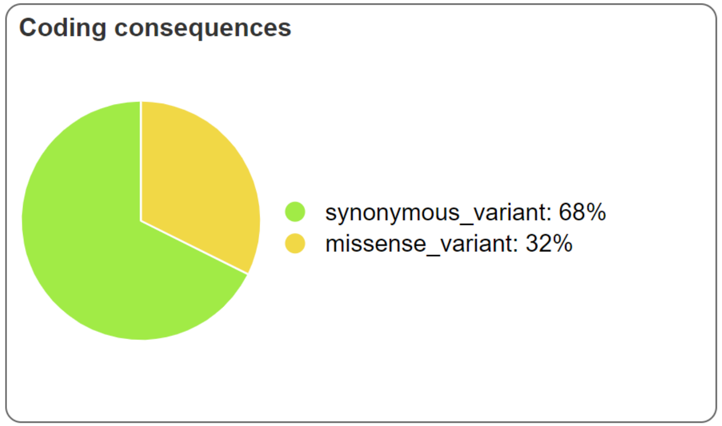

2e. Target region 4 variant summary

Category	Count
Variants processed	3008
Variants filtered out	0
Novel / existing variants	76 (2.5) / 2932 (97.5)
Overlapped genes	3
Overlapped transcripts	11
Overlapped regulatory features	29

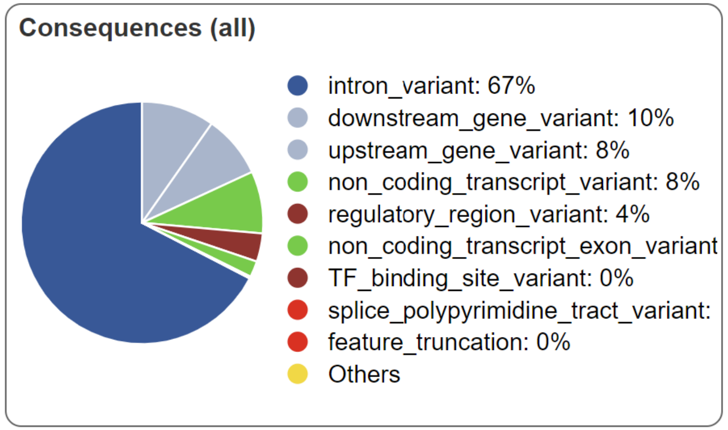

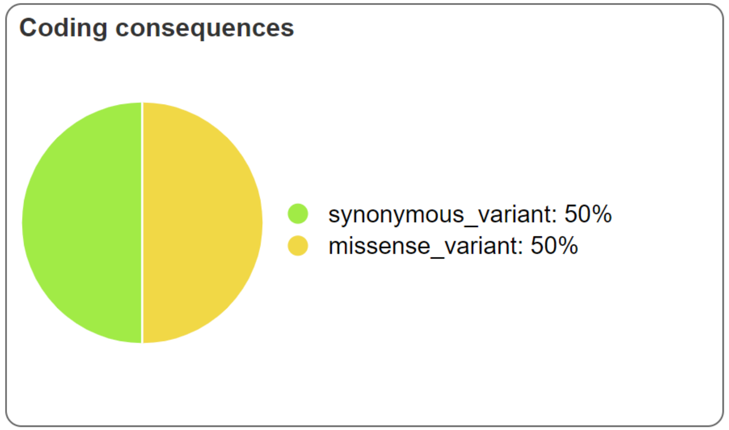

2f. Target region 5 variant summary

Category	Count
Variants processed	899
Variants filtered out	0
Novel / existing variants	44 (4.9) / 855 (95.1)
Overlapped genes	1
Overlapped transcripts	48
Overlapped regulatory features	28

Consequences (all)

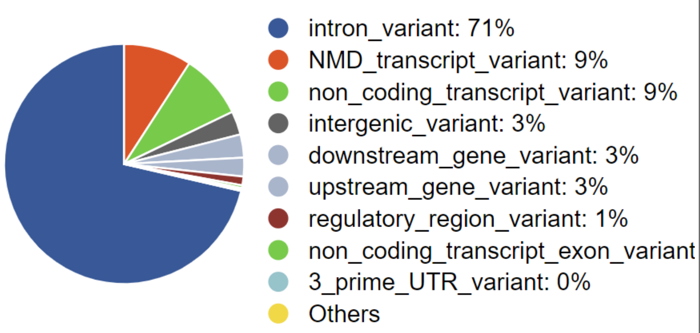

Coding consequences

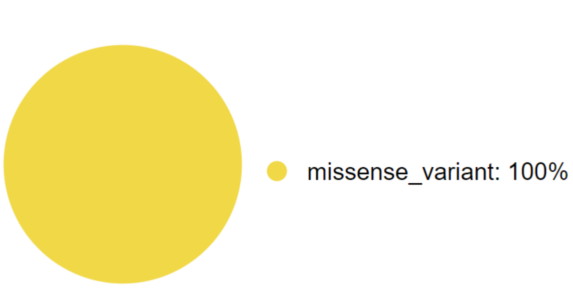

2g. Target region 6 variant summary

Category	Count
Variants processed	3610
Variants filtered out	0
Novel / existing variants	80 (2.2) / 3530 (97.8)
Overlapped genes	4
Overlapped transcripts	4
Overlapped regulatory features	33

Consequences (all)

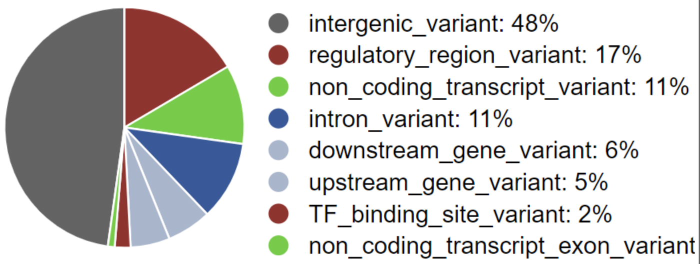

2h. Target region 7 variant summary

Category	Count
Variants processed	3265
Variants filtered out	0
Novel / existing variants	71 (2.2) / 3194 (97.8)
Overlapped genes	1
Overlapped transcripts	5
Overlapped regulatory features	11

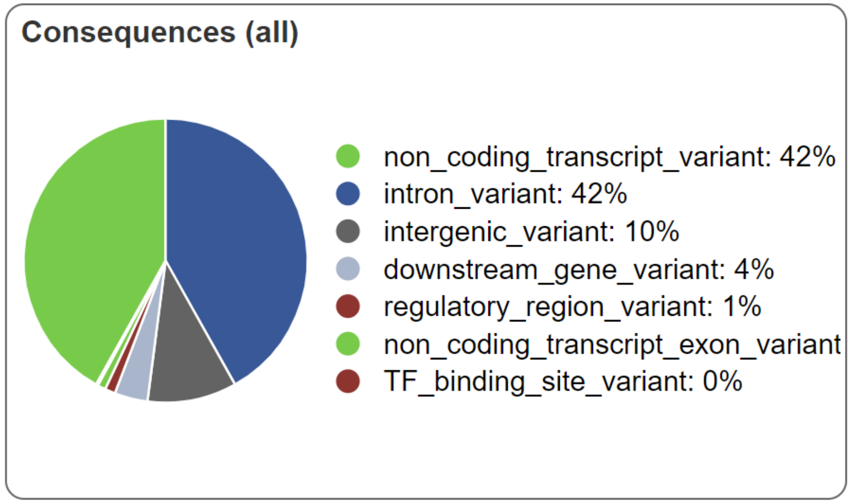

2i. Target region 8 variant summary

Category	Count
Variants processed	3440
Variants filtered out	0
Novel / existing variants	65 (1.9) / 3375 (98.1)
Overlapped genes	4
Overlapped transcripts	40
Overlapped regulatory features	18

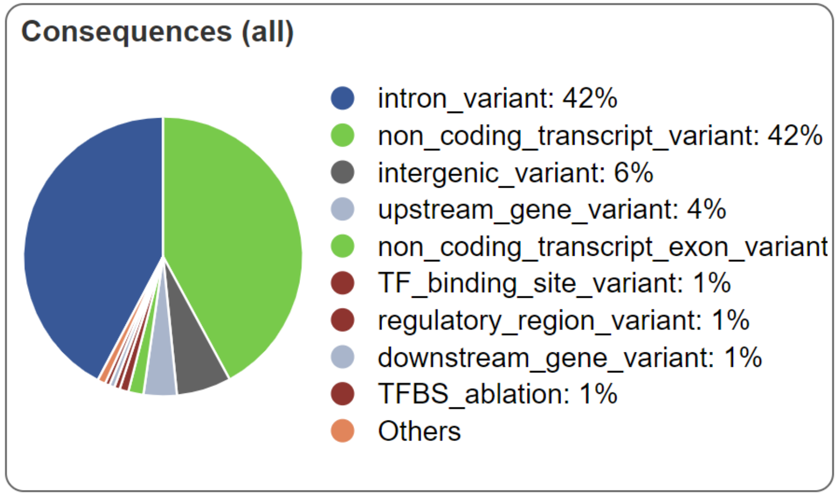

Supplement: Supplementary file 2 — Supplementary Material 2 [file 12864_2024_11172_MOESM2_ESM.pdf]
