# Supplementary material for: Nanopore sequencing as a novel method of characterising anorexia nervosa risk loci
Source: BMC Genomics. 2024 Dec 31;25:1262. doi: 10.1186/s12864-024-11172-7 (PMC11687000; doi:10.1186/s12864-024-11172-7)

Supplementary Figure 3. TopLD linkage disequilibrium graphs calculated from the ANGI GWAS summary statistics via TopLD and plotted with Locus Zoom-like R tool. Target regions 1 to 8 are depicted in figures a-h, respectively. The boxes with dashed outlines indicate the genomic regions used for strong LD filtering in variant prioritization. The blue circles overlapping the gene maps below the LD plots indicate the approximate position of the prioritised variants.

a) Target region 1 (chr3:48580820-48780819)

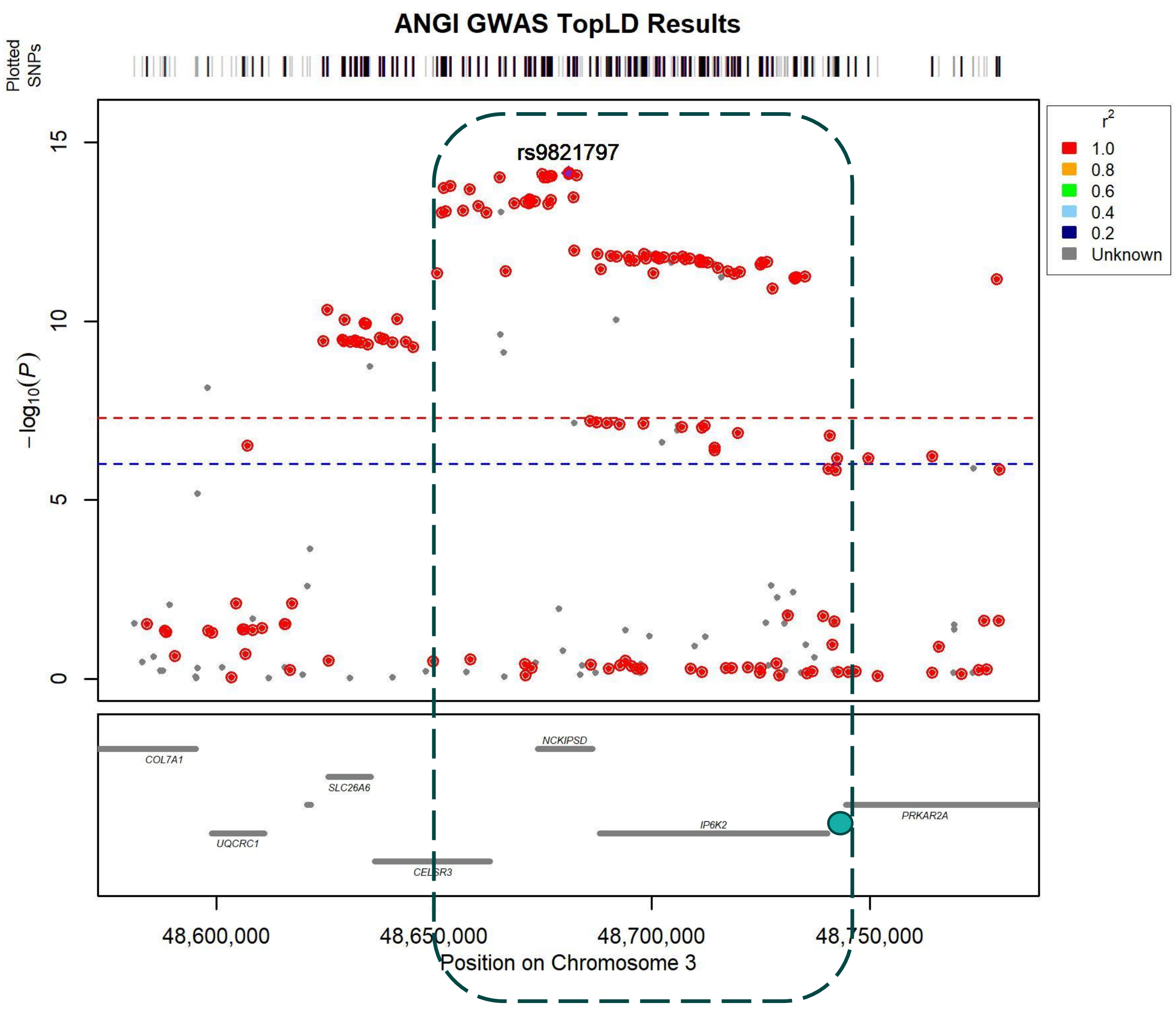

b) Target region 2 (chr11:115126236-115326235)

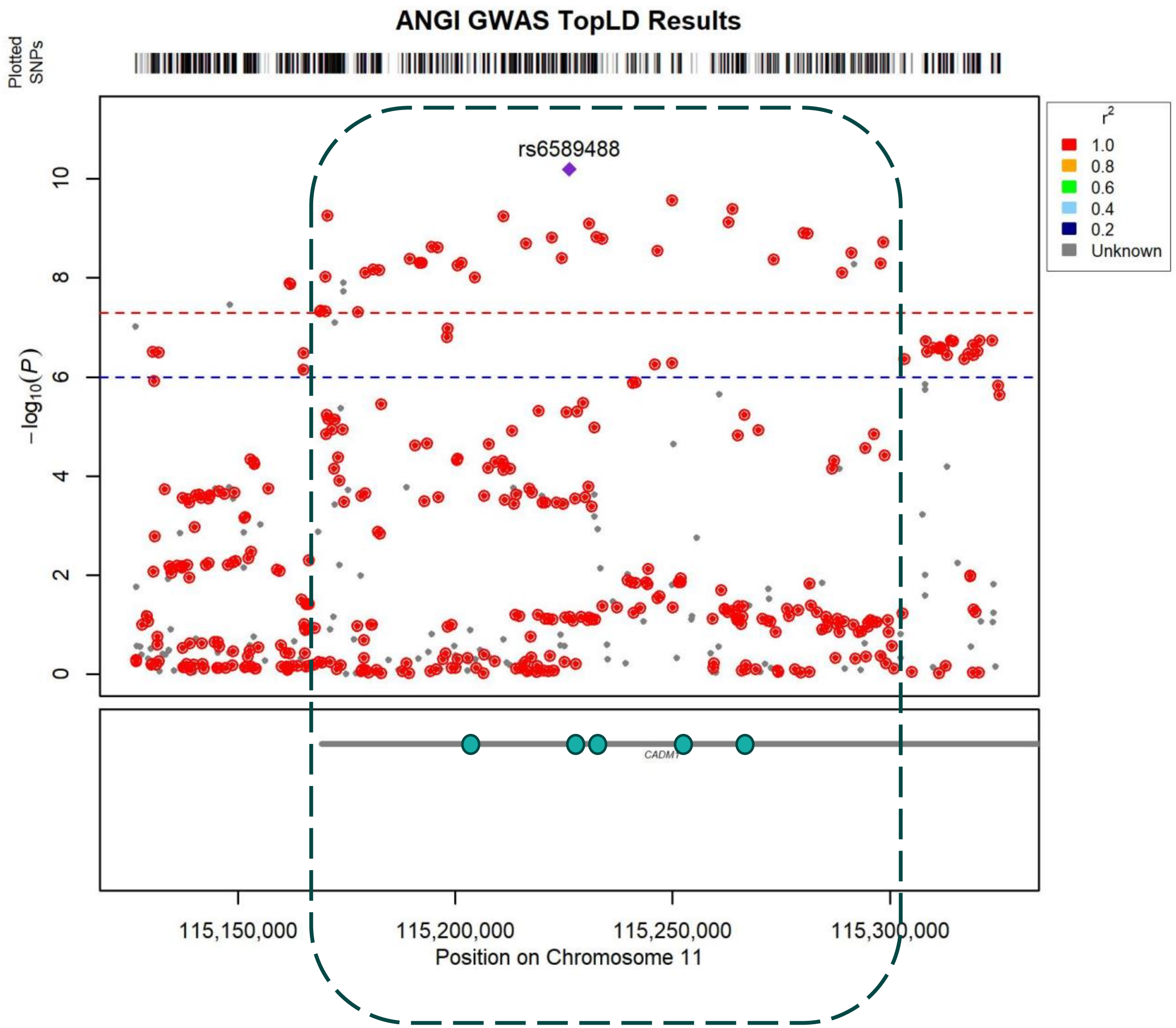

c) Target region 3 (chr2:53712676-53912676)

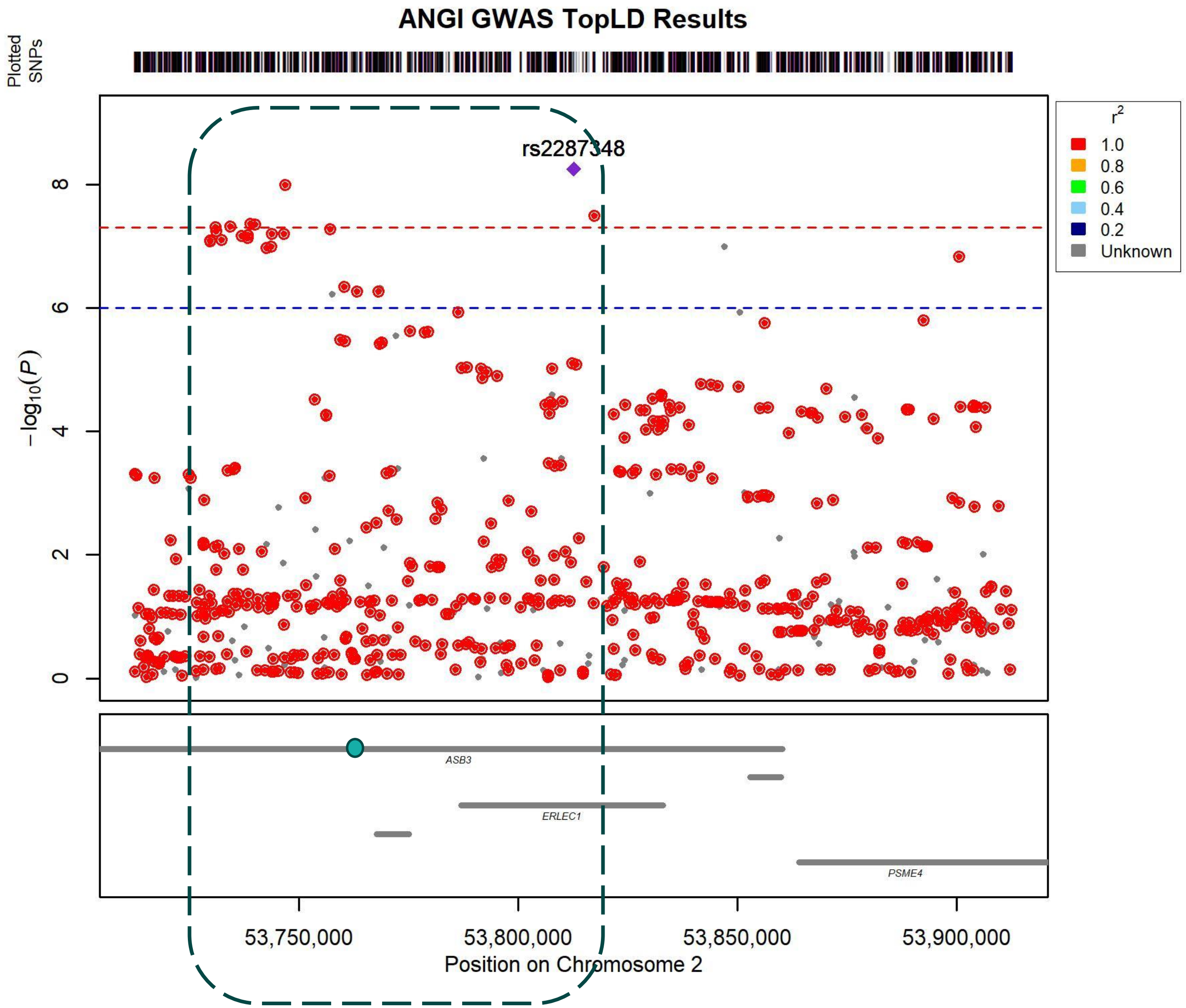

d) Target region 4 (chr10:129550500-129750499)

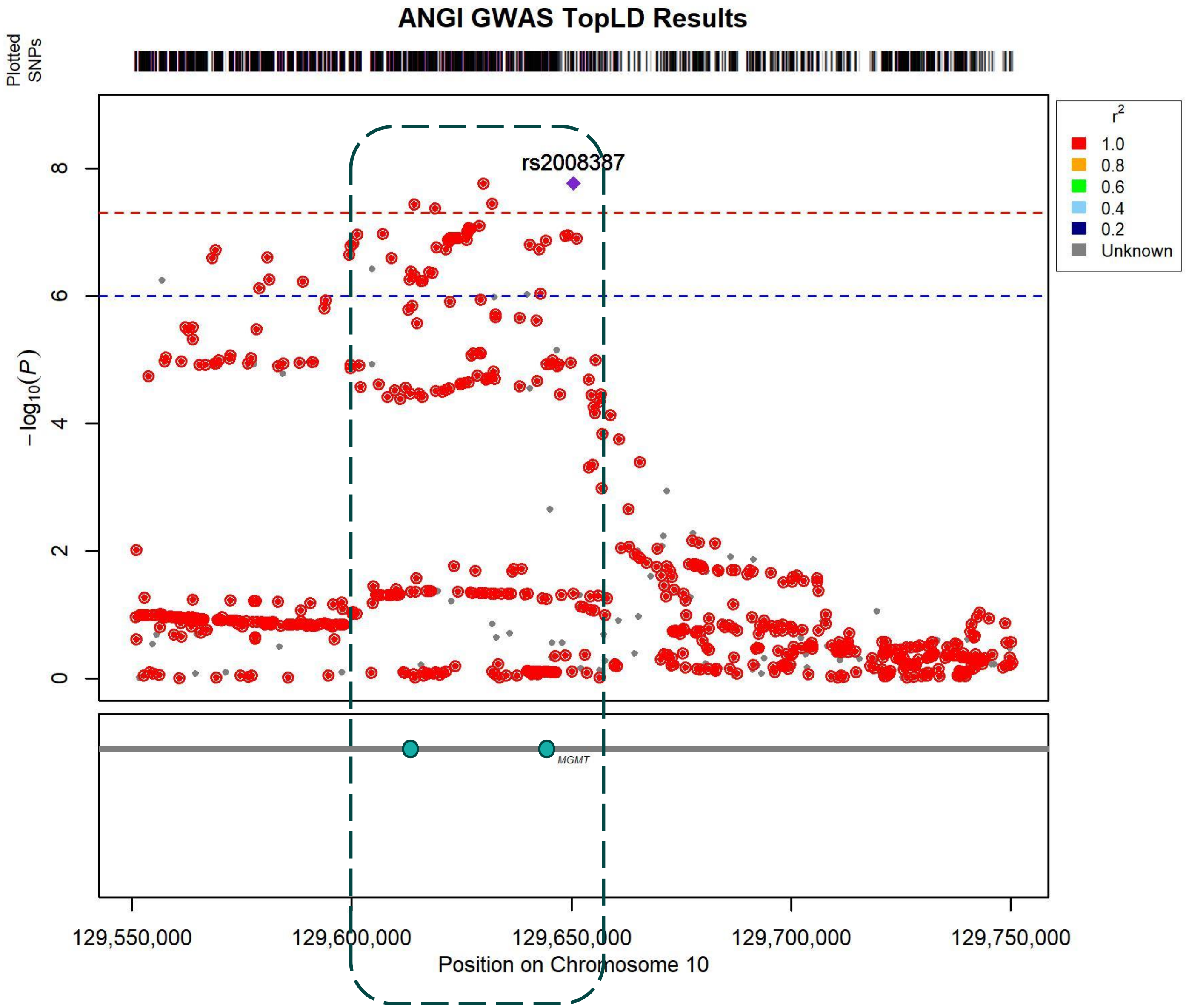

e) Target region 5 (chr3:70870599-71070598)

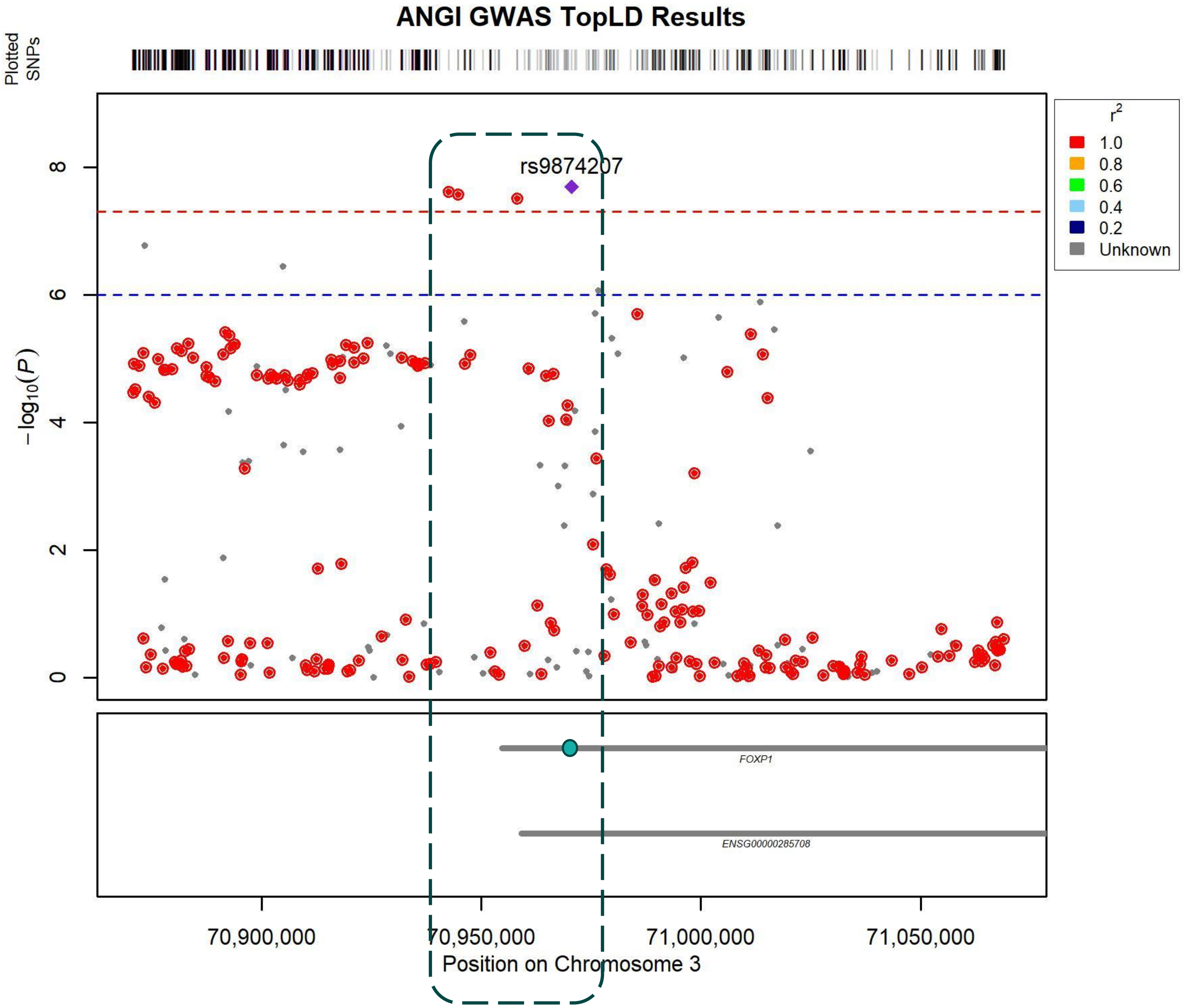

f) Target region 6 (chr1:96335899-96535898)

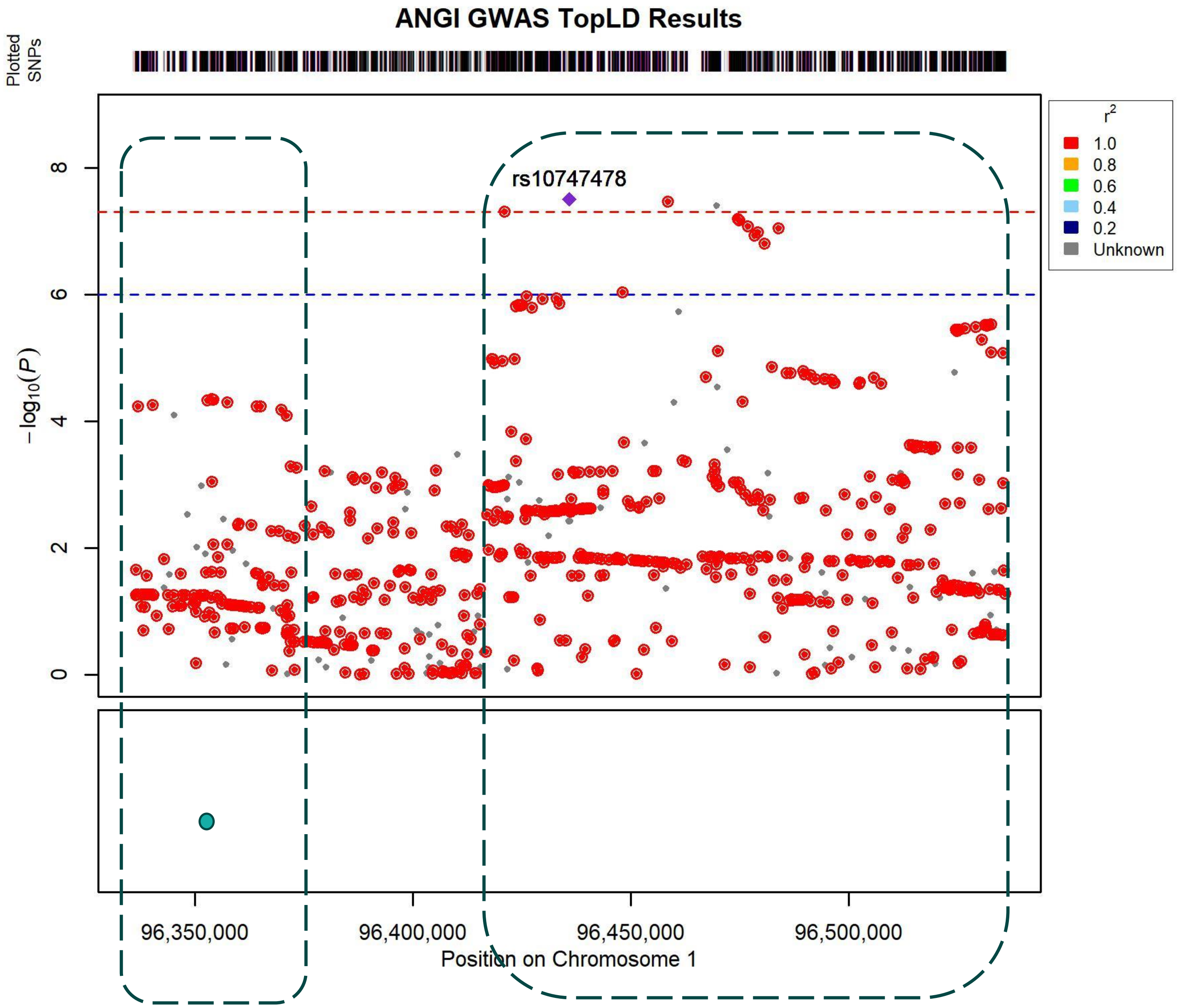

g) Target region 7 (chr5:24981736-25181735)

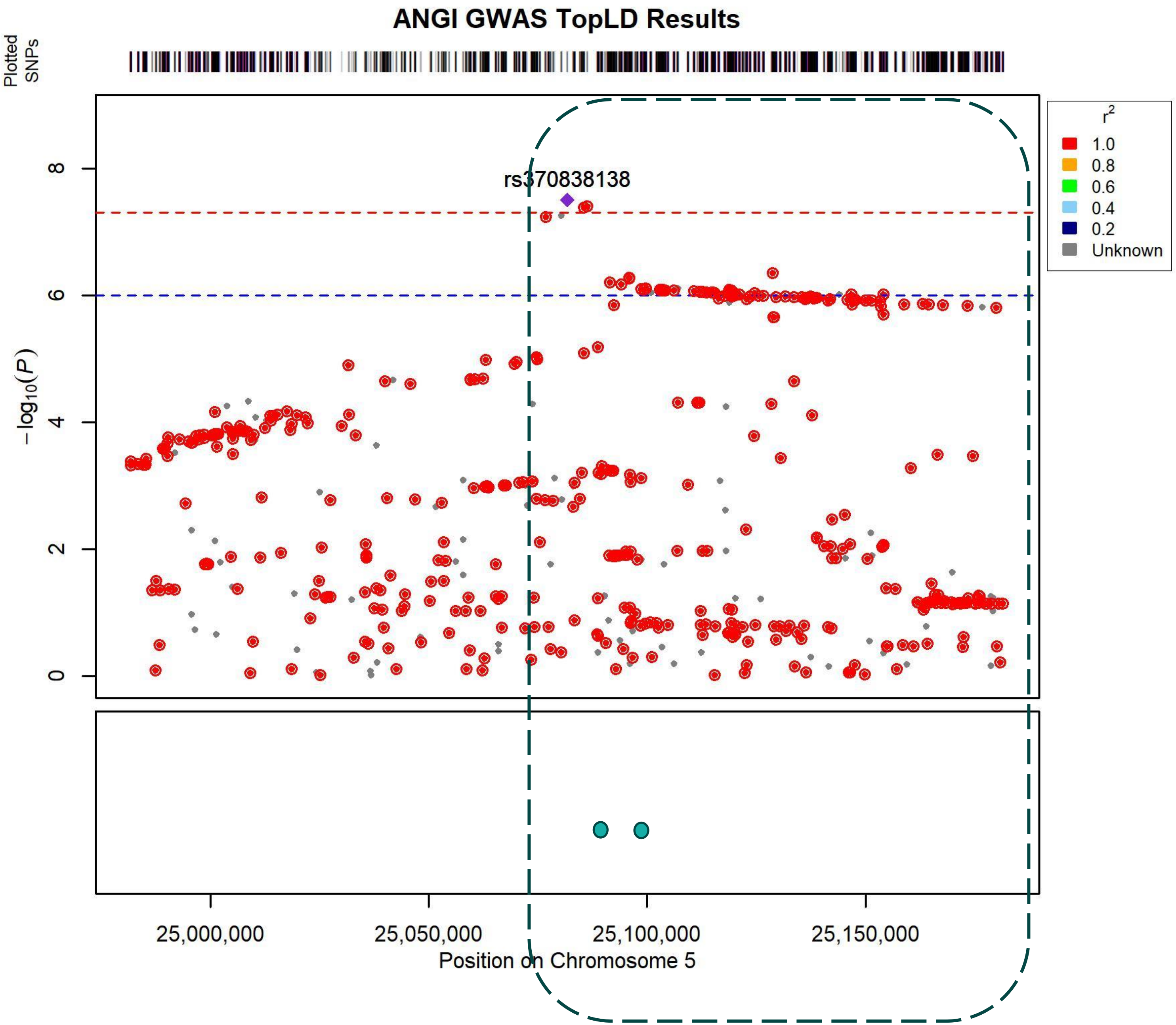

h) Target region 8 (chr3:94786263-94986262)

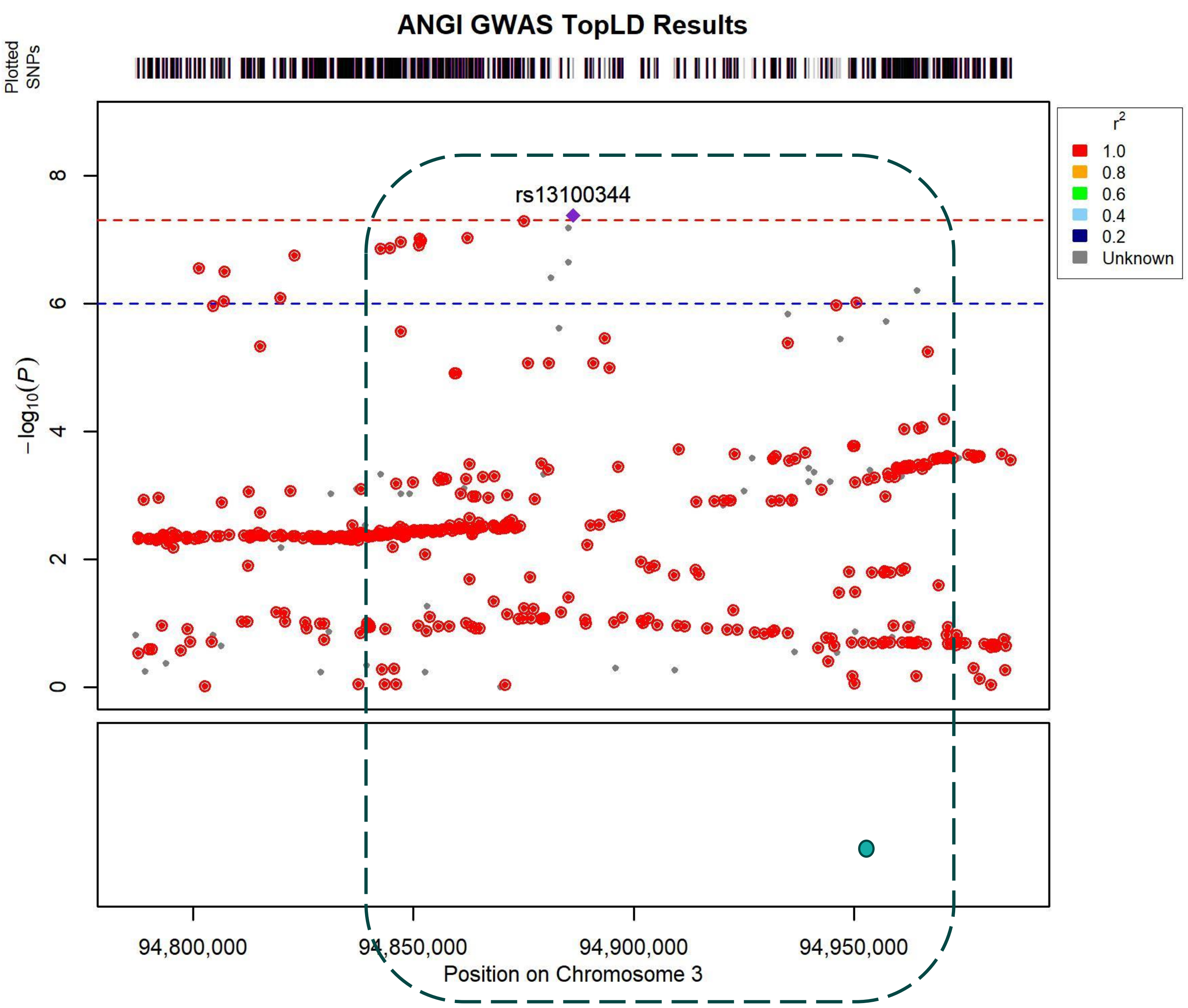

Supplement: Supplementary file 3 — Supplementary Material 3 [file 12864_2024_11172_MOESM3_ESM.pdf]
